# Confidence-based laboratory test reduction recommendation algorithm

**DOI:** 10.1186/s12911-023-02187-3

**Published:** 2023-05-10

**Authors:** Tongtong Huang, Linda T. Li, Elmer V. Bernstam, Xiaoqian Jiang

**Affiliations:** 1 School of Biomedical Informatics, UTHealth, Houston, TX USA; 2 Department of Pediatric Surgery, McGovern Medical School, UTHealth, Houston, TX USA; 3 Division of General Internal Medicine, Department of Internal Medicine, McGovern Medical School, UTHealth, Houston, TX USA

**Keywords:** Lab test reduction, Deep learning, Confidence based

## Abstract

**Background:**

We propose a new deep learning model to identify unnecessary hemoglobin (Hgb) tests for patients admitted to the hospital, which can help reduce health risks and healthcare costs.

**Methods:**

We collected internal patient data from a teaching hospital in Houston and external patient data from the MIMIC III database. The study used a conservative definition of unnecessary laboratory tests, which was defined as stable (i.e., stability) and below the lower normal bound (i.e., normality). Considering that machine learning models may yield less reliable results when trained on noisy inputs containing low-quality information, we estimated prediction confidence to assess the reliability of predicted outcomes. We adopted a “select and predict” design philosophy to maximize prediction performance by selectively considering samples with high prediction confidence for recommendations. Our model accommodated irregularly sampled observational data to make full use of variable correlations (i.e., with other laboratory test values) and temporal dependencies (i.e., previous laboratory tests performed within the same encounter) in selecting candidates for training and prediction.

**Results:**

The proposed model demonstrated remarkable Hgb prediction performance, achieving a normality AUC of 95.89% and a Hgb stability AUC of 95.94%, while recommending a reduction of 9.91% of Hgb tests that were deemed unnecessary. Additionally, the model could generalize well to external patients admitted to another hospital.

**Conclusions:**

This study introduces a novel deep learning model with the potential to significantly reduce healthcare costs and improve patient outcomes by identifying unnecessary laboratory tests for hospitalized patients.

**Supplementary Information:**

The online version contains supplementary material available at 10.1186/s12911-023-02187-3.

## Introduction

Laboratory over-utilization is common, especially in the United States [1, 2]. Unnecessary blood draws waste resources and may harm patients by contributing to iatrogenic anemia [3]. Ideally, we want to minimize laboratory utilization while obtaining necessary information [1, 4].

Trivial rule-based approaches are not generalizable to all laboratory tests due to the complexity and heterogeneity of patient conditions. Unlike these methods, machine learning approaches can offer more flexibility to define necessary and sufficient conditions of an unnecessary laboratory test. Recently, researchers have used machine learning to identify unnecessary laboratory tests. Most approaches leverage time series prediction methods to take advantage of previous patient information, such as autoregressive models [5], mixed-effect models [6], and traditional recurrent neural networks (RNN) [7, 8]. Previous studies that identify unnecessary laboratory tests fall into two categories: *information gain,* and observability learning.

*Information gain* methods measure whether certain laboratory tests yield informative values (i.e., large information gain). These approaches require an exact definition of unnecessary laboratory tests. Roy et al. [5] identify laboratory tests in the normal range as low-yield laboratory tests, but they ignore events where the laboratory value changes from the normal to abnormal range; however, transitions such as these are likely to be clinically relevant events. Cismondi et al. [9] dichotomize laboratory tests into “information gain” or “no information gain” categories based on both normality and dropping levels. Aikens et al. [10] consider laboratory stability using both absolute value and standard deviation changes. Their models recommend eliminating laboratory tests that have little information gain. However, these algorithms lack confidence estimates to measure the reliability of predictions. Metrics that summarize the confidence of a prediction are important adjuncts for clinicians using the algorithm.

*Observability learning* approaches estimate the need for a lab to be checked in actual practice. A missing observation means that the laboratory test was not checked by the physician. Yu et al. [11, 12] used a two-layer long short term memory (LSTM) network with multiple fully-connected layers to estimate the observability of the next laboratory test. The min–max loss function increases prediction accuracy as the likelihood of observability (i.e., the necessity of conducting the next laboratory test) increases. Their first approach [11] has a narrow definition of the ground truth, which aims to predict the change rate for laboratory values. The second approach [12] extends the previous model by using multitask learning mechanisms to include predictions for abnormality (i.e., laboratory values beyond the normal range) and transition (i.e., laboratory values change from the normal to the abnormal range or vice versa). However, such recommendation algorithms are highly dependent on actual physician practices, which might not be optimal for laboratory test reduction algorithms. Relying on past practices might not be applicable to different or complicated clinical situations. Observability learning models, which work to predict the need for a test, are likely to recommend eliminating laboratory tests with low prediction confidence. We believe that, in addition to considering the future laboratory value, a better strategy of laboratory test reduction may be to simultaneously consider laboratory tests that can be confidently predicted.

To address the underlying issues above, we propose a novel approach that is distinct from the existing *information gain* and *observability learning* studies. Our work is designed to reduce unnecessary hemoglobin (Hgb) tests based on the following conservative assumptions. Sequential Hgb levels within the normal range imply that the patient is stable [13, 14]. We further define that the Hgb test is “stable” when its immediate value does not change from normal to abnormal.

We explore approaches that enable our algorithm to make safe and confident predictions with features embedded in the algorithm. First, we introduce an *outcome-level* safety assurance, which uses a conservative (‘safe’) definition of unnecessary Hgb laboratory tests. Specifically, we define ‘safe’ Hgb tests as ones that are predicted to remain stable and normal. If our model predicts that the Hgb level will remain stable and normal, we will recommend eliminating the next Hgb test. Second, we will estimate the confidence of predicting Hgb test values as a *sample-level* safety assurance. Existing algorithms that estimate prediction confidence in a post-hoc manner, such as Monte Carlo Dropout (MC Dropout) [15] and Bayesian Neural Networks (BNN) [16] are computationally expensive and require generating multiple predictions for each input during testing. To enhance the computation efficiency, we estimate the confidence for predicting Hgb at a specific time based on all available time-series data by introducing a selection predictor in the neural network architecture [17]. During training, the model ignores certain samples whose inclusion would decrease the performance of the model. During testing, the selection predictor does not make recommendations if the selection confidence is below a certain threshold.

Our main contribution is the integration of the confidence-based selection process for candidate samples into both the training and testing phases of the algorithm, instead of estimating prediction confidence in a post-hoc manner. We do not address clinical adoption issues such as specific confidence thresholds in this paper, which are outside the scope of our methodology investigation.

## Method and materials

### Dataset

#### Local hospital data

To validate the model’s performance internally, we utilized local hospital data to predict Hgb based on data from a variety of hospitalized patients. The data was obtained from a large urban hospital system in the southern United States and encompassed 75,335 distinct inpatient encounters that occurred between 1/1/2020 and 12/6/2020. Because the model requires learning from previous observations of laboratory data and we did not recommend omitting tests for patients who only had one Hgb test, we excluded 8,528 encounters with only one Hgb test. We also eliminated 4,328 encounters with systolic blood pressures less than 90 mmHg to focus on patients who were hemodynamically stable, resulting in a final cohort of 62,479 unique encounters. This included 804 pediatric encounters (age < 18). The demographics are summarized in S Table 1. To train and test the model, we partitioned the entire cohort into 80% training data and 20% test data. Data splitting was performed at the encounter level, which means that each encounter was treated as a separate instance, and all observations of laboratory data associated with that encounter were grouped together.

#### MIMIC III data

To validate the model’s generalizability externally, we trained with local hospital data (Section Local hospital data) and tested using an unrelated critical care dataset. We collected 55,340 encounters from MIMIC (Medical Information Mart for Intensive Care) III (recorded in 2001—2012). After applying the exclusion criteria (Section Local hospital data), we had 46,847 unique encounters, including 1,176 pediatric encounters. The demographics are summarized in S Table 1. We took the entire MIMIC III cohort in the external validation set.

#### Data description

The dataset consists of 12 commonly ordered laboratory tests and 5 relevant vital signs (i.e., peripheral pulse rate, diastolic blood pressure, systolic blood pressure, respiratory rate, and SpO2 percent), as well as patient demographics (i.e., gender, race, and age), time differences (i.e., time from last observation), Hgb value changes, and missing value indicators. Specifically, the laboratory tests include:

- Electrolytes: Na (sodium), K (potassium), Cl (chloride), HCO3 (serum bicarbonate), Ca (total calcium), Mg (magnesium), PO4 (phosphate)
- Renal function: BUN (Blood Urea Nitrogen), Cr (creatinine)
- Complete Blood Count (CBC): Plt (platelet count), WBC (white blood count), Hgb (hemoglobin, and we predicted Hgb prescribed in the CBC panel)

These 12 laboratory tests are often ordered together in panels. Each panel and its associated vital signs were considered as the unit of observation. We included all laboratory data performed during the same encounter, regardless of whether it was collected before or after admission.

### Data preprocessing

For each encounter, we organized laboratory test results into a consecutive sequence in temporal order, with several laboratory tests conducted in the same hour aggregated into a laboratory draw. If a laboratory test appeared more than once at the same hour, we averaged the test results. The laboratory draws for each encounter was constrained to start with the draw where the first Hgb results were recorded, and end with the draw where the last Hgb result was recorded. To make the parameter space manageable, we capped the total length of laboratory draws to 30 timestamps based on the histogram of visit times (i.e., the number of timestamped laboratory records for encounters, see S Fig. 3). We padded zeros to the end of these laboratory draws if the sequence length was less than 30. To account for the patient's condition, we integrated vital signs with the laboratory draw based on the event time. We calculated average values when more than one vital sign was reported during the same hour. Additionally, we included patient demographics, involving gender, race, and age information. The normal range varies depending on gender and age (Table 1).

**Table 1:** Hgb normal range stratification table

Age	Hgb LBNR (g/dL)	Hgb UBNR (g/dL)
6 months to 6 years	10.5	14.5
7—12 years	11.0	16.0
Adult Women	12.0	16.0
Adult Men	14.0	18.0

LBNR means the lower bound of the normal range. UBNR means the upper bound of the normal range. Our experiments only considered Hgb LBNR to identify normality and stability

In our basic setup, we represented each encounter record of size 30 × 39, with each timestamp having a dimension of 1 × 39. The 39 dimensions correspond to 12 laboratory tests, 5 vital signs, 3 demographic variables, time difference, Hgb value change, and 17 missing indicators of laboratory and vital features (S Fig. 4). This representation allowed us to incorporate all relevant information for each encounter into our model.

### Notation

In our model, we used a number of symbols to represent important concepts related to the multivariate time series data that we analyzed. These symbols are summarized in Table 2. For each encounter, we had a multivariate time series data of length $T$. As observations were not necessarily made at regular intervals, hence, the timestep $t$ simply indexed the sequence of observations.

**Table 2:** Nomenclature table

Symbol	Explanation
$X$	Input features (including laboratory values, vital signs, demographics, time differences in terms of hours, and observation indicators)
$O$	Observation indicator. It is included in input features$X$. (If the laboratory sample is observed,${o}_{t}=1$; otherwise, ${o}_{t}=0$)
$T$	The total number of timesteps. In a time sequence, a record of a laboratory group occurs at one timestep. The maximum number of timesteps is 30
$P$	The selection probability (If the Hgb is selected, ${p}_{t}=1$; otherwise, ${p}_{t}=0$)
$Y$	Normality label (If the Hgb value is above the LBNR, ${y}_{t}=1$; otherwise, ${y}_{t}=0$)
$Z$	Stability label (If the Hgb value does not transit from normal to abnormal, ${z}_{t}=1$; otherwise, ${z}_{t}=0$)
$V$	Hemoglobin value
$\tau$	The selection threshold. Its default value is 0.5. (If predicted selection probability ${p}_{t}>\tau$, the corresponding Hgb sample is selected as a candidate for reduction; otherwise, the Hgb sample is not a candidate for reduction)

The feature measurements $X$ represent a group of input features that we used to generate predictions. These features include laboratory values, vital signs, Hgb value changes, demographics, time differences, and observation indicators. Hgb normality labels $Y$, Hgb stability labels $Z$, and Hgb values $V$ served as gold standards to measure our model’s prediction performance. Specifically, Hgb normality and stability are determined by its normal range (Table 1). To measure the confidence in predicting the next Hgb test, our model also predicted the selection probability $P$. The normality ${y}_{t}$, stability ${z}_{t}$, values ${v}_{t}$, and selection probabilities ${p}_{t}$ represented the outcome realized at timestep $t$.

To handle missing values in the dataset, we used observation indicators denoted as $O$. If an encounter had observed data (i.e., a Hgb test drawn and resulted) at a timestep $t$, we denoted the observation indicator ${o}_{t}=1$; Otherwise, we denoted ${o}_{t}=0$.

We used a sigmoid function to output predictions for normality $Y$, stability $Z$, and selection probability $P$ in a range of $[\mathrm{0,1}]$. Predictions for $V$ were restricted to be positive. We used a threshold of 0.5 to make cutoffs for classifications of normality, stability, and selection probability. In the test stage, the selection threshold was adjustable, and we set values in the range of $\tau \in [\mathrm{0.05.0}.95]$.

### Prediction tasks

Our confidence-based selection model has four predictive tasks: selection probability ${p}_{t}$, Hgb normality ${y}_{t}$, Hgb stability ${z}_{t}$, and Hgb value ${v}_{t}$. Specifically, predicting Hgb normality ${y}_{t}$ and Hgb stability ${z}_{t}$ are responsible for *outcome-level* safety assurance, and predicting selection probability ${p}_{t}$ is responsible for *sample-level* safety assurance.

Selection probability ${p}_{t}$ measures the confidence of the model’s predictions for the next Hgb. Prediction confidence represents predictability, that is, the reliability of Hgb predictions estimated by the model. Based on Hgb tests that are confidently predicted, our model can make more accurate predictions of normality and stability. The model selected Hgb candidates with high prediction confidences, whose ${p}_{t}$ is greater than a selection threshold $\tau$.

For the Hgb normality ${y}_{t}$ task, a predicted value above the lower bound of the normal range (LBNR) indicated a normal (i.e., ${y}_{t}=1$) Hgb test, while a value below LBRN indicated an abnormal (i.e., ${y}_{t}=0$) test. The study assumes that Hgb results exceeding the upper bound of the normal range (UBNR) are relatively uncommon (e.g., polycythemia) or irrelevant (e.g., indicate dehydration or chronic hypoxia that are usually monitored using other modalities and were excluded from our analysis) to our analysis. Notably, the majority of clinical cases focus on dropping Hgb (e.g., bleeding), and only 0.37% of Hgb results in our samples were above the UBNR. In our population-driven model, instead of using a uniform LBNR over the entire population, we defined normal Hgb based on age and gender (Table 1).

For the Hgb stability ${z}_{t}$ task, a predicted value was considered stable (i.e., ${z}_{t}=1$) if it remained within the normal range and did not shift from normal to abnormal. Specifically, only a decreasing drop from normal to abnormal was considered for this task. Agreement between normality and stability predictions reinforces the confidence of the overall prediction.

The Hgb value ${v}_{t}$ was an auxiliary task aimed at improving the model’s primary prediction tasks – Hgb normality and stability. We expected that Hgb value predictions with confident normality and stability predictions would also closely approximate the actual values.

Given an encounter’s input features $X$, we defined the ground truth of unnecessary Hgb tests as the intersection of the following conditions: $\{{X|y}_{t}=1\}\cap \{X|{z}_{t}=1\}\cap \{X|{p}_{t}>\tau \}$. This means that if the next Hgb value was predicted to be normal and stable among the selected candidates, the model would recommend a ‘safe’ reduction.

### Clinical significance and implementation

We envision that the model could be integrated into the electronic health record (EHR) system, and the output of the model could be presented to the clinician through a user interface within the EHR. In practice, the model could present recommendations to the clinician regarding the necessity of a laboratory test for a given patient. To implement the model, we first choose an acceptable confidence level to determine the subset of laboratory tests to be selected. The selected tests will be considered high-confidence candidates. We recommend canceling pending laboratory tests if it is predicted to be normal and stable among the selected candidates; otherwise, we recommend clinicians check the following laboratory tests. The clinician would retain the final decision-making authority based on their assessment of the patient’s clinically relevant observations that may not be captured by our model (e.g., surgical procedures, hemoptysis).

It is worth noting that our proposed model should be used as a decision-support tool, rather than a replacement for clinical judgement. The system allows the clinician to override the model’s recommendation and order a laboratory test if they deem it necessary based on their clinical judgement.

### Feature processing

This section introduces our methods to handle missing data and random mask corruption. We incorporated relational positional time embeddings to replace absolute one-dimensional time differences. Since time embeddings have little impact on improving model performance in our experiment, we discuss details of this approach in S Text.

#### Missing data handling

In our study, we utilized 12 common laboratory tests as features, but some tests were not conducted in the same time window. This was due to the fact that no patient had all laboratory tests drawn at the same time. We treated these unmeasured laboratory tests as missing values, which are denoted as ${v}_{t}=0$. Previous work [18] has demonstrated that deep learning models, such as long short term memory (LSTM) networks, can effectively handle missing data by integrating an additional indicator ${o}_{t}=0$ for these missing values ${v}_{t}=0$. Thus, our model utilized two vectors (${o}_{t}$,${v}_{t}$) instead of a single vector of observed lab test values, enabling the model to discern which observations were missing. This so-called zero imputation strategy can handle missing data implicitly by considering feature correlations.

#### Random mask corruption

During the training stage, our approach employed a random mask corruption method to simulate the impact of laboratory test reduction on future predictions. This was achieved by introducing a hypothetical scenario where a laboratory test is reduced at time $t$, making its observed value unavailable for predicting future tests at time $t+1$. To simulate this scenario, we randomly corrupted 10% of observed inputs (${o}_{t}=1$) by setting their corresponding value as ${v}_{t}=0$ in future predictions. The motivation behind this approach was based on prior research [12], which indicated that random mask corruption is an effective way to enhance model performance under the assumption of laboratory test reduction. We still considered the prediction errors of these corrupted Hgb values from time $t-1$ to $t$, but did not use them to make future predictions. During the testing stage, we did not introduce any corruption and simply changed ${v}_{t}=0$ for lab tests that were recommended for reduction with respect to future predictions.

### Development of confidence-based deep learning approach

**Fig. 1:**
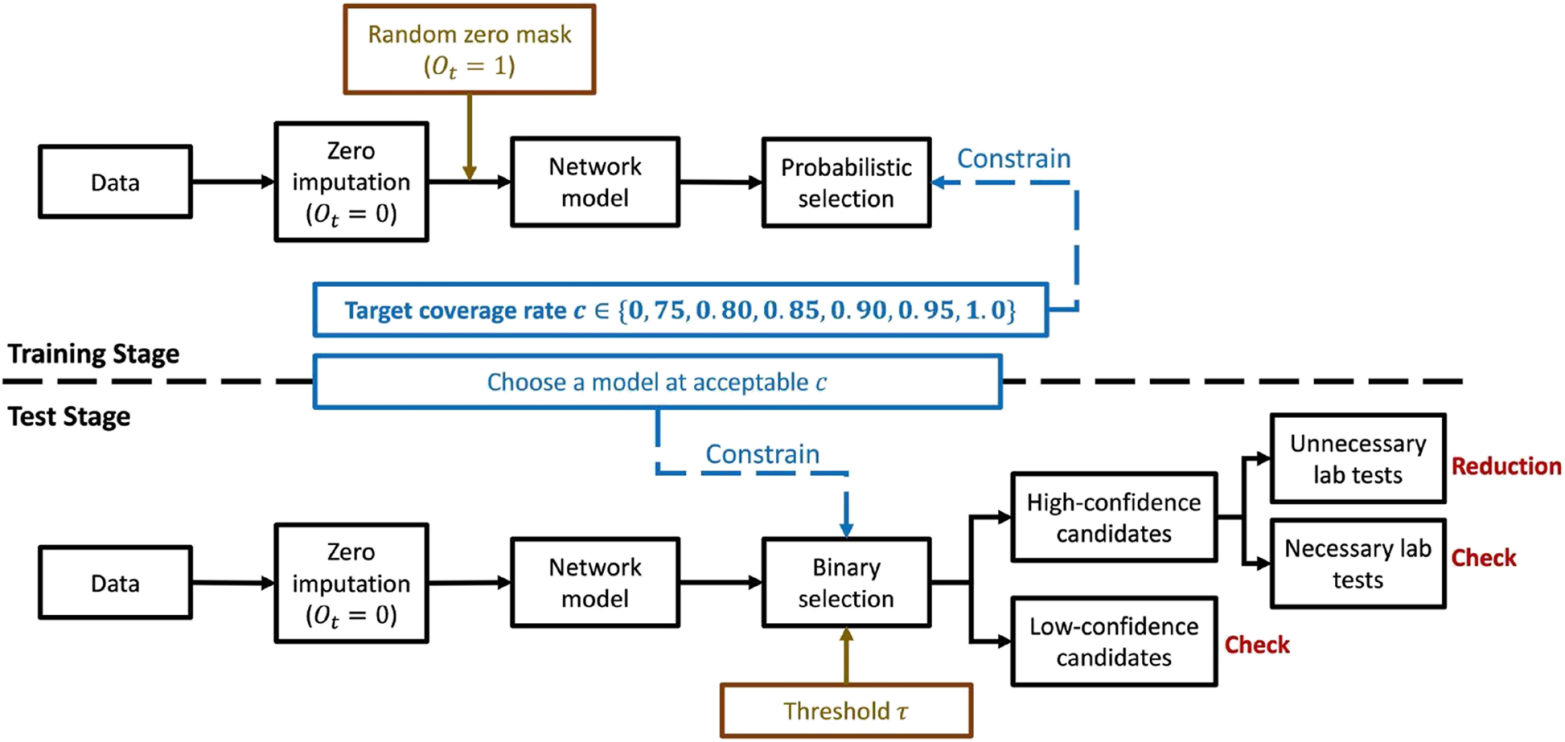
Block diagram of candidate selection. (1) We imputed zeros for missing features before processing the network model. (2) In the training stage, we inserted a random zero mask for existing laboratory values. The network model predicted selection probabilities for individual laboratory tests. Thus, the model ignored some samples, whose inclusions were considered to decrease performance. Each model was trained under one target coverage rate that constrained the actual proportion of selected laboratory tests. Intuitively, the lower coverage rate means that selections are stricter. (3) We chose a model at an acceptable coverage rate in the test stage. A threshold $\tau$ was used to determine whether individual laboratory tests were selected. The selected tests were considered high-confidence candidates. The model recommended canceling pending laboratory tests if predicted values satisfied two joint conditions: a. High confidence; b. Unnecessary (i.e., predicted to be stable and remain normal)

**Fig. 2:**
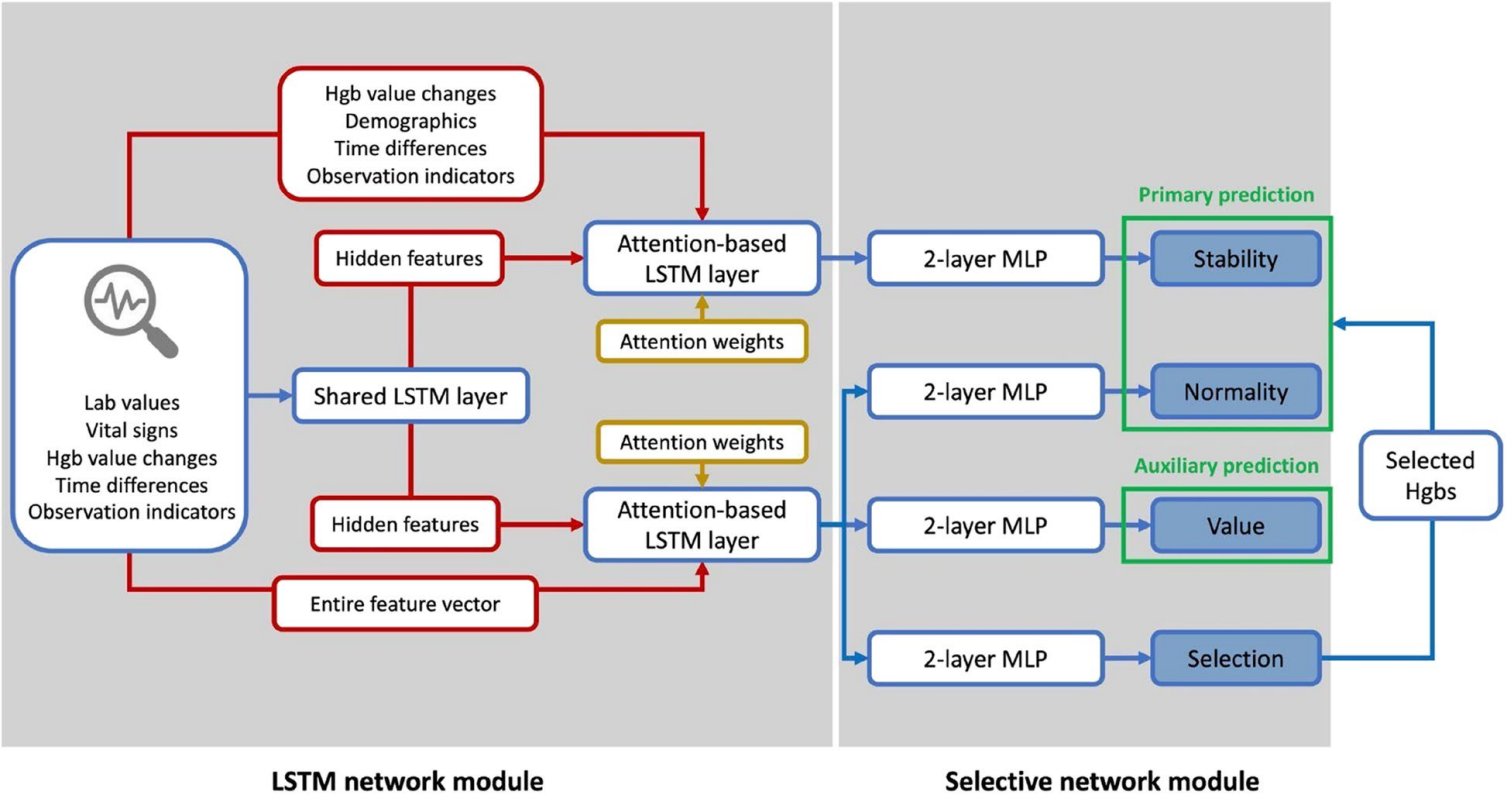
Model Architecture Framework. In the LSTM network module, the shared LSTM layer received all input features, and outputs hidden features that contained general information derived from original data. The attention-based LSTM layer augmented input embeddings by concatenating hidden features and duplicating original features. One attention-based layer learned a subset of features for the following stability predictor. The other attention-based layer learned entire feature vectors to obtain complicated information for the following normality, value, and selection predictor. In the selective network module, we had four 2-layer MLP predictors to make task-specific predictions for Hgb stability, Hgb normality, Hgb value, and selection probability in parallel. Stability and normality predictors were treated as primary predictions that focus on selected Hgb samples. The value predictor served as the auxiliary prediction that covered all Hgb samples, including the non-selected ones

The workflow of the confidence-based candidate selection is demonstrated in Fig. 1. The zero imputation and random zero mask mechanism were described in Section Feature Processing. The architecture of the network model was described in Section Feed-forward Attention LSTM and Section Network Model Architecture and Fig. 2. The loss function to constrain the selection of laboratory test candidates was described in Section Loss Function with Reject Optimization.

#### Feed-forward attention LSTM

A time-aware attention mechanism [19] was used to extract essential features from the input sequence over time. First, the LSTM layer generated a sequence of hidden vectors $h$. Second, for each timestep $t$, the learnable function $a$ computed the hidden vector ${h}_{t}$, then produced an encoded embedding ${e}_{t}$. We computed a probability weight ${a}_{t}$ using a softmax function over the entire time sequence. Finally, the context vector $c$ was computed as a weighted sum of the hidden vectors ${h}_{t}$.

1 $${e}_{t}=a({h}_{t})$$

2 $${a}_{t}=exp({e}_{t})/\sum\limits_{k=1}^{T}exp({e}_{k})$$

3 $$c=\sum\limits_{t=0}^{T}{a}_{t}{h}_{t}$$

As a result, the attention mechanism enabled the model to distribute weights over the entire time period, which smoothly adjusted the impacts of long-time memories for irregular time sequences.

#### Network model architecture

We proposed a novel confidence-based deep learning approach using LSTM and the selective neural network under multitask learning strategies. The main idea is to select a subset of Hgb candidates with high prediction confidence so that the selected Hgb candidates are more likely to make correct classifications of normality and stability. The architecture of our model is depicted in Fig. 2. The input features were first fed into the LSTM network module, which consists of a common feed-forward LSTM layer to capture the shared information and two attention-based LSTMs for task-specific learning. Our model architecture was inspired by a previous multitask learning approach [20]. The shared LSTM layer accepted all features (i.e., laboratory values, vital signs, patient demographics, Hgb value changes, time differences, and observation indicators) as inputs, and optimized hidden states at every timestep. Demographic features were duplicated and placed in every timestamp. The two attention-based LSTM layers, which shared parameters, capture task-specific information. In the attention-based LSTM layer, input embeddings were augmented by concatenating the hidden features of the shared LSTM layer and original data $X$.

The stability predictor was implemented following the first attention-based LSTM layer. We used Hgb values to measure the stability of future Hgb tests, and this first layer learned a subset of features, including Hgb value changes, patient demographics, time differences, and observation indicators. On the other hand, the normality, value, and selection predictor was implemented following the second attention-based LSTM layer. These three prediction tasks require complex features and may influence each other [11]. This second layer learned entire feature vectors, including laboratory values, vital signs, patient demographics, Hgb value changes, time differences, and observation indicators.

The downstream selective network module employed multilayer perceptrons (MLPs) with ReLU activation, each followed by a final task-specific prediction. Each MLP predictor consisted of two fully-connected layers. The selection MLP predictor estimated the likelihood of choosing the Hgb test as a high-confidence sample [17], and its details will be explained in the next section. The normality and stability MLP predictors were responsible for the primary predictions of selected Hgb candidates. The value MLP predictor made the auxiliary prediction that covered all Hgb samples, including Hgb tests that were not selected by the model. The auxiliary prediction was introduced to prevent overfitting (i.e., the model chooses non-representative samples to only benefit normality and stability prediction). The Hgb value optimization used the entire dataset to evaluate the loss in order to ensure the generalizability of normality and stability predictors.

#### Loss function with reject optimization

We considered the problem of selective prediction in the laboratory reduction network and leveraged the integrated reject mechanism developed in SelectiveNet [17]. It is a deep learning architecture that is optimized for selecting samples that maximally benefit model predictions. Based on our assumption that the dataset contains a proportion of Hgb outliers, such a specialized rejection model only considers a proportion of samples and filters out low-confidence ones. The approach proposes a loss function that enforces the coverage constraint using a variant of the Interior Point Method (IPM) [21]. The selection head outputs a single probabilistic value ${p}_{t}$ using a sigmoid activation. At a certain timestep $t$, the selective network module achieves LSTM hidden features, then predicts Hgb normality ${f}_{Y}({x}_{t})$ and stability ${f}_{Z}({x}_{t})$ if and only if the selection probability ${p}_{t}$ exceeds a user-defined threshold $\tau$ (Table 2); otherwise, the model rejects the prediction tasks of normality and stability for ${x}_{t}$. Given the selection loss ${L}_{f}$ in Eq. (4), the performance of the selective algorithm is measured by the likelihood of normality $r({f}_{Y})$, the likelihood of stability $r({f}_{Z})$, and a quadratic penalty function $\psi (a)$.

4 $${L}_{f}=r({f}_{Y})+r({f}_{Z})+\lambda \psi (c-\phi (P))$$

5 $$\psi (a)=max(0,a{)}^{2}$$

where $c$ is the customized target coverage (i.e., the expected proportion of samples to be considered eligible for reductions, for which the model will predict), $\lambda$ is a penalty parameter to control the weight of the regularization. The likelihood of normality $r({f}_{Y})$ and the likelihood of stability $r({f}_{Z})$ are determined by the normality loss $L({f}_{Y}({x}_{t }),{y}_{t})$ and stability loss$L({f}_{Z}({x}_{t }),{z}_{t})$, respectively.

6 $$r({f}_{Y})=(1/T\sum\limits_{t=0}^{T}L({f}_{Y}({x}_{t }),{y}_{t }){p}_{t})/\phi (P)$$

7 $$r({f}_{Z})=(1/T\sum\limits_{t=0}^{T}L({f}_{Z}({x}_{t}),{z}_{t}){p}_{t})/\phi (P)$$

where

8 $$\phi (P)=1/T\sum\limits_{t=0}^{T}{p}_{t}$$

In Eq. (9) and (10), the normality loss $L({f}_{Y}({x}_{t }),{y}_{t})$ and stability loss $L({f}_{Z}({x}_{t }),{z}_{t})$ are both calculated using the binary cross-entropy, where $\sigma$ denotes the sigmoid function that converts predictions into probabilistic values.

9 $$L({f}_{Y}({x}_{t }),{y}_{t})={y}_{t}log(\sigma ({f}_{Y}({x}_{t })))+(1-{y}_{t})log(1-\sigma ({f}_{Y}({x}_{t })))$$

10 $$L({f}_{Z}({x}_{t }),{z}_{t})={z}_{t}log(\sigma ({f}_{Z}({x}_{t })))+(1-{z}_{t})log(1-\sigma ({f}_{Z}({x}_{t})))$$

Additionally, we handled Hgb value predictions ${f}_{V}({x}_{t})$ as the auxiliary task using a standard mean squared error (MSE) loss function. Auxiliary predictions were used to connect the selection loss ${L}_{f}$ by accounting for all samples. Otherwise, normality loss and stability loss only consider optimizing predictions of selected samples, which might cause the overfitting issue. Thus, the overall loss function $L$ is a combination of the selection loss ${L}_{f}$ and the auxiliary loss ${L}_{aux}$ as follows:

11 $$L=\alpha {L}_{f}+(1-\alpha ){L}_{aux}$$

where $\alpha$ is the selection weight which lies in $[\mathrm{0,1}]$, and

12 $$L_{aux}=1/T{\textstyle\sum_{t=0}^T}(f_V(x_t)-v_t)^2$$

#### Training and evaluation design

When internally evaluating the model, the algorithm was trained and tested with local hospital data (Section Local hospital data). When externally evaluating the model, the algorithm was trained with local hospital data and tested with MIMIC III data (Section MIMIC III data). We present model performance tested on the local hospital data in Sections Label Prediction Performance, Value Prediction Performance, and Selection Performance, and on MIMIC III in Section Model Performance on MIMIC III.

In the training stage, we conducted random mask corruption (Section Random mask corruption) to transform some observations into zeros in order to simulate the impact of recommended lab test reduction. In the test stage, we either converted omitted laboratory values to zeros (in reduction evaluation) or kept the original laboratory values (in no-reduction evaluation).

- *Training protocol:* In the training stage, input features $X$ were fed into the network model at a fixed length $T$. The prediction at every timestep $t$ depends on the observations from all previous timesteps. We trained separate models at the target coverage rate $c=\{0.75, 0.8, 0.85, 0.9, 0.95, 1.0\}$, which reflects the expected proportion of laboratory candidates that are selected for possible reduction.
- *Reduction evaluation (practical setting):* In the test stage, we simulated dynamic laboratory reduction during the evaluation process. Starting at the initial timestep $t=0$, the model was fed initial inputs ${x}_{0}$. For the following timesteps $t>0$, we conducted stepwise reduction estimation. If the model estimated the next Hgb to be normal and stable, which yielded a recommendation to omit that test, the next Hgb input would be set at a zero value. The reduction evaluation process iterated until the last timestep.
- *No-reduction evaluation (idealized setting):* Like the training stage, the second evaluation protocol performed a fixed evaluation process. At every timestep $t$, the model obtained all input features from the previous timesteps. In this process, we always used full observation to make future predictions, and no lab test was ever reduced in the prediction process. Model robustness can be estimated from the gap between *reduction* and *no-reduction* evaluations.

## Results

### Baseline comparison

To evaluate the impact of different components on the overall performance of the proposed model, we conducted an ablation study for our proposed model (Table 3). Specifically, we investigated the performance of the model without time embeddings (S Text), which treated time differences as a one-dimensional vector. We also examined the model without attention weights, which omitted the attention mechanism in any LSTM layer, and the model without confidence selections, which did not predict selection probabilities and excluded the corresponding loss function. To establish a baseline, we compared the network structure of our model with that of vanilla LSTM, which was derived from a state-of-art *observability learning* model targeting at reducing unnecessary laboratory tests [12].

**Table 3:** Comparison with baseline models at an optimal coverage (c = 0.85)

Benchmark	Target Coverage	Model Coverage	Normality					Stability				
			**Prev**	**AUC**	**Acc**	**Prec**	**AUPRC**	**Prev**	**AUC**	**Acc**	**Prec**	**AUPRC**
**Our Model**	0.85	83.80%	15.25%	**95.89%**	**93.17**%	74.38%	**80.05%**	97.40%	**95.94%**	97.41%	97.46%	99.89%
**Without Time Embedding**	0.85	85.28%	15.64%	95.61%	92.85%	**74.63%**	79.53%	97.99%	94.92%	**98.01%**	**98.01%**	99.89%
**Without Attention**	0.85	84.75%	15.99%	92.68%	90.11%	85.53%	72.99%	97.95%	95.44%	97.96%	97.99%	**99.90%**
**Without Confidence Selection**	-	-	20.20%	90.91%	85.19%	59.28%	63.92%	90.90%	94.80%	93.48%	95.68%	99.46%
**Vanilla LSTM**	-	-	20.20%	88.40%	82.64%	58.10%	61.04%	90.90%	94.80%	92.91%	94.65%	99.47%

Bold numbers denote the best performances over the evaluation metric, and underlined numbers denote the second-best performance over the evaluation metric

The results of the ablation study demonstrate that our model outperformed the vanilla LSTM, which was attributed to the integration of attention and confidence selection mechanisms. The attention mechanism improved normality AUC (i.e., the AUROC of normality predictions) by 3.21% and AUPRC by 7.06%, while the confidence selection mechanism improved normality AUC by 4.98% and AUPRC by 16.13%. Moreover, the time embedding mechanism also contributed to improving normality accuracy, and achieved comparable stability accuracy to the full model.

### Label prediction performance

**Fig. 3:**
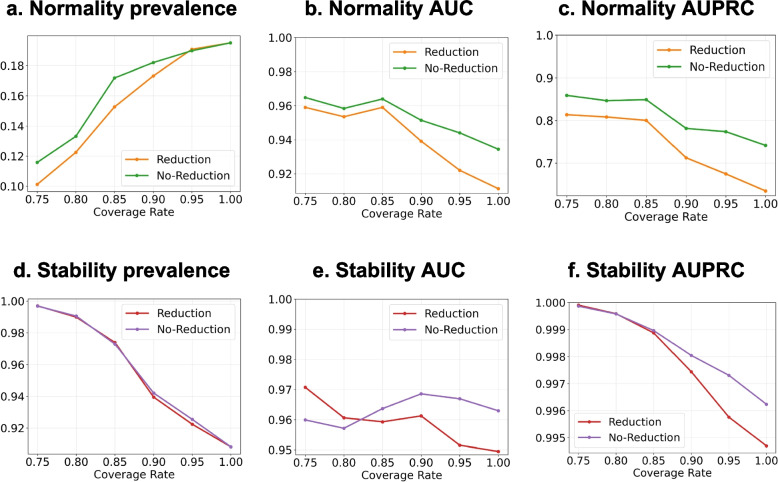
Model performance at multiple selection coverages under reduction and no-reduction evaluation. The prevalence refers to the proportion of positive samples (i.e., normal and stable Hgbs) in the selected Hgb candidates. The normality AUC and stability AUC refer to the AUROC for the normality prediction and stability prediction, respectively. The selection threshold $\tau$ is 0.5. The coverage rate refers to the expected proportion of Hgb samples to construct the model

We conducted multiple experiences to demonstrate the effectiveness of our proposed confidence-based selection model in predicting normality and stability labels. Our model selected high-confidence samples using a default selection threshold of $\tau =0.5$ and a coverage rate $c$ (Section Loss Function with Reject Optimization). A trade-off exists between being too strict on prediction confidence, which would reduce the selection size and affect the model’s generalizability, and being too lenient, which would include unreliable samples. We evaluated our model under reduction evaluation and no-reduction evaluation metrics (Section Training and Evaluation Design), and the comparison results are shown in Fig. 3 and numerical results in Table 4 and S Table 4.

**Table 4:** Model performance by selection coverages and reduction rate

Target Coverage	Model Coverage	Reduction Rate	Normality					Stability				
			**Prev**	**AUC**	**Acc**	**Prec**	**AUPRC**	**Prev**	**AUC**	**Acc**	**Prec**	**AUPRC**
**0.75**	77.92%	3.08%	10.09%	95.92%	95.16%	78.28%	81.52%	99.70%	96.91%	99.70%	99.70%	99.99%
**0.80**	81.64%	4.94%	12.24%	95.34%	94.49%	82.33%	80.78%	98.99%	95.99%	99.01%	99.02%	99.96%
**0.85**	83.80%	9.91%	15.25%	95.89%	93.17%	74.38%	80.05%	97.40%	95.94%	97.41%	97.46%	99.89%
**0.90**	89.84%	11.71%	17.28%	93.89%	89.47%	64.89%	71.12%	93.94%	96.06%	94.84%	97.21%	99.74%
**0.95**	96.91%	16.89%	19.07%	92.24%	86.76%	60.99%	67.56%	92.25%	95.19%	94.22%	95.77%	99.58%
**1.0**	99.99%	17.48%	19.51%	91.10%	85.64%	59.18%	63.43%	90.82%	94.94%	93.43%	95.36%	99.47%

The model coverage rate refers to the actual proportion of Hgb samples considered by the model; the reduction rate refers to the proportion of Hgb samples recommended for reduction among the entire Hgb test dataset; Prev indicates prevalence, which denotes the proportion of normal/stable Hgbs in selective candidates; AUC indicates area under the ROC curve; Acc indicates accuracy; Prec indicates precision; AUPRC indicates the precision-recall curve

To differentiate the concepts, we defined “model coverage rate” as the actual proportion of Hgb samples considered by the model, while “target coverage rate” as the customized coverage constraint $c$. Our results (Table 4, S Table 4) demonstrated that “model coverage rates” were close to “target coverage rates”, and they had less than 2% of differences in all settings, showing that the model is enforcing the target coverage rate.

We employed AUC and AUPRC as performance measures since the data distribution was skewed, with only 19.5% of labs being normal and only 9.2% transitioning from normal to abnormal. The results demonstrated that our model achieved normality AUCs (Fig. 3 b) at over 90%, and stability AUCs (Fig. 3 e) at over 94%, even in the extreme coverage case at $c=1$. Notably, despite setting $c=1$ (Table 4), our model did not consider all Hgb predictions as good candidates for reduction, in which our results represented 99% of Hgb samples eligible for reduction recommendation. We observed that our model reduced 9.91% Hgb tests at a “target coverage rate” of 0.85 (Table 4), achieving a good tradeoff between performance and reduction. Most AUCs and AUPRCs decreased as the “target coverage rate” increased, except for stability AUCs, which did not drop at a steady rate because the proportion of stable samples was much larger than unstable ones, resulting in the mean probability bias favoring stable lab tests [22]. Our model achieved comparable performances over no-reduction evaluations (Fig. 3), demonstrating its robustness in practical settings when some input laboratory values are missing due to previous reductions. Moreover, as the “target coverage rate” increased, the normality prevalence (Fig. 3. a) increased linearly, while the stability prevalence (Fig. 3. c) decreased linearly. This suggested that our higher-confidence model tends to select more samples from the dominating class of labels (i.e., abnormal Hgbs and stable Hgbs) to obtain higher accuracy while recognizing minor labels (i.e., normal Hgbs and unstable Hgbs).

### Value prediction performance

We evaluated the consistency between predicted values and predicted normality, conducted under the reduction evaluation. Our aim was to determine whether the predicted values were consistent with the predicted normality, with a particular focus on the expected location of normal Hgb samples above the LBNR.

**Fig. 4:**
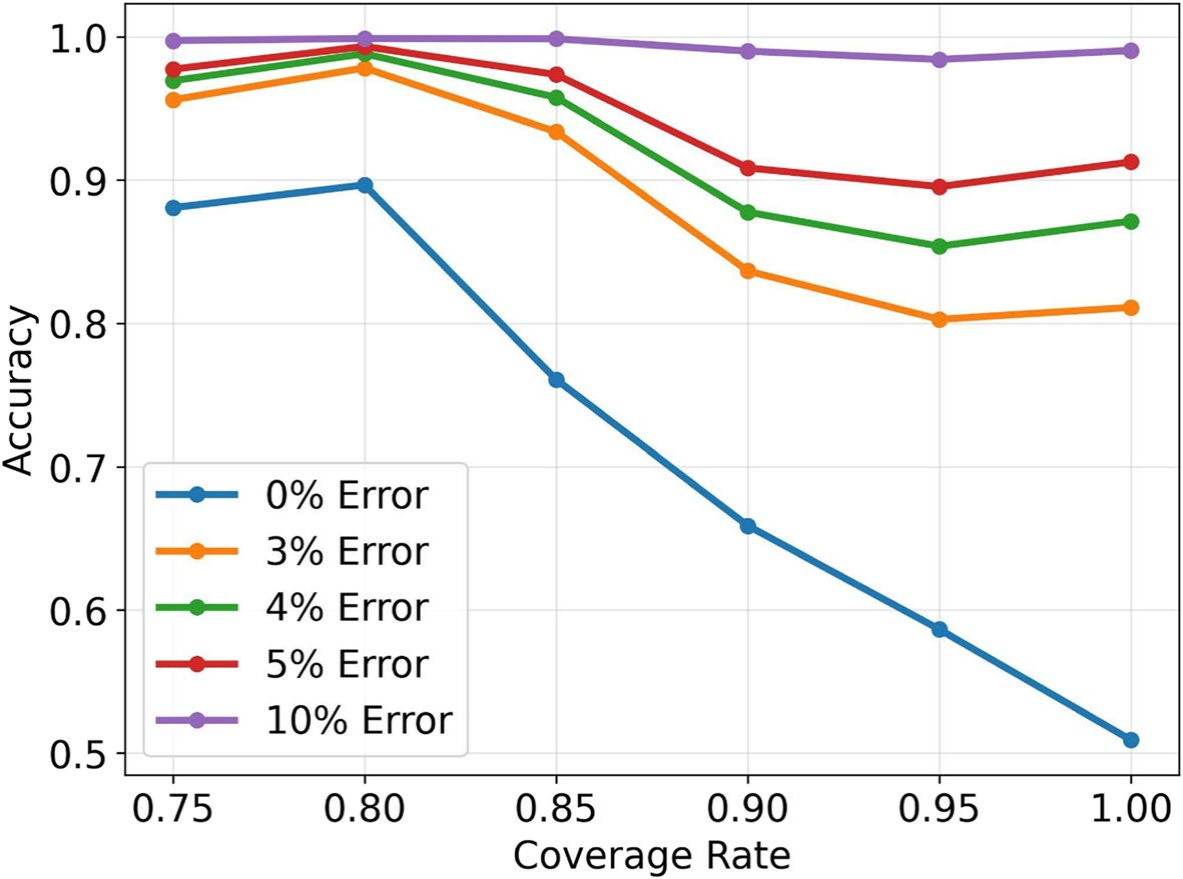
Consistency between predicted values and predicted normality. The objective was to measure the consistency between predicted values and predicted normality. The “normality accuracy of predicted values” was defined as the percentage of predicted values with ${f}_{V}({x}_{t})>=b$, where $b$ is the value of the LBNR, on Hgb samples with ${f}_{Y}({x}_{t})=1$. We considered a tolerable boundary to be m% lower than the LBNR for predicted normal Hgbs

Figure 4 indicated that our proposed multitask learning framework was successful in leveraging the auxiliary task of value prediction to support the primary prediction tasks of normality and stability prediction. Specifically, we observed that, among Hgbs predicted to be normal, more than 90% of corresponding values were predicted to be above the LBNR with a tolerable error of 3% at “target coverage rates” less than 0.85, and with a tolerable error of 5% at almost all “target coverage rates”. These findings suggested that our model’s predicted values were consistent with the expected normality predictions, validating the effectiveness of our proposed framework.

Furthermore, our results also suggested that the “target coverage rate” has a significant impact on recognizing the normality of predicted values. As the coverage rate increased, the accuracy of normality prediction showed more variability at error rates of 0–10%, indicating that the low-confidence model had great variability in its value predictions.

### Selection performance

We present the evaluation of our model’s classification accuracy with different selection thresholds $\tau \in [\mathrm{0.05,0.95}]$ (with intervals at 0.05) under laboratory reductions. During training, we set the selection threshold at $\tau =0.5$ and used the different values in the test phase. The “target coverage rate” was set to 0.85.

**Fig. 5:**
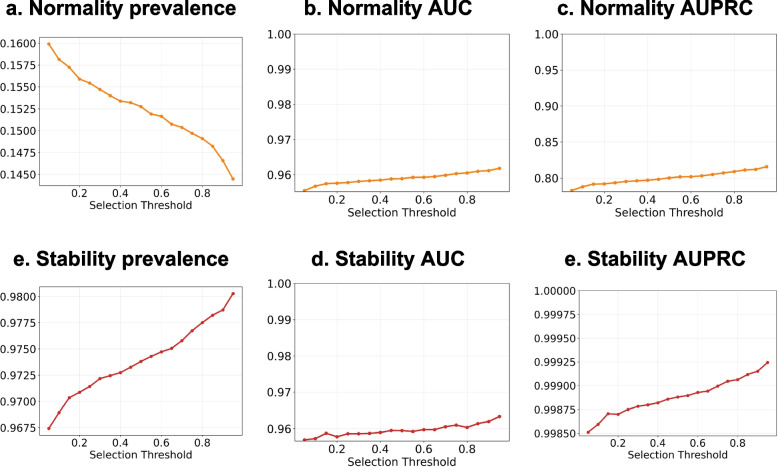
Model performances using different selection thresholds for reduction evaluation. The range of our selection threshold is $\tau \in [0.05, 0.95]$ with intervals at 0.05

Figure 5 illustrated that the performance curves fluctuated at nearby selection thresholds, but the trend remained consistent and increased as the threshold value increased. With a higher threshold, the model selected fewer normal Hgbs and more stable Hgbs, resulting in higher confidence in classifications. When the threshold $\tau$ was above 0.5, our model achieved normality predictions over 95.8% AUC and 80.0% AUPRC. Similarly, stability predictions achieved more than 95.9% AUC and 99.8% AUPRC. These results demonstrated that our model’s performance was robust to the selection threshold and could achieve high accuracy with different values of the threshold.

### Model performance on MIMIC III

**Fig. 6:**
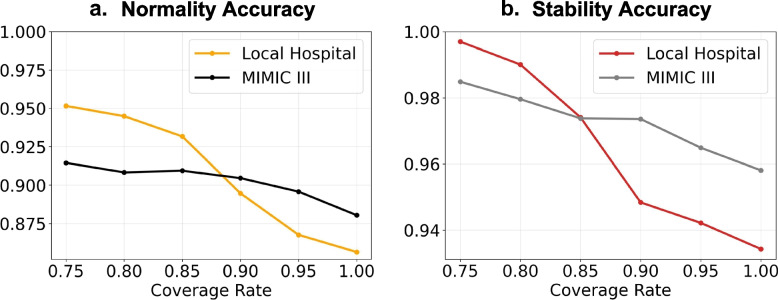
Comparing model accuracy when evaluating local hospital data and MIMIC III data. The selection threshold $\tau$ is 0.5

We conducted an external evaluation of our model using the MIMIC III dataset to assess its generalizability in predicting Hgb values for patients admitted to another healthcare institution. Our model demonstrated normality prediction accuracy of over 88% and stability prediction accuracy of over 95% (Fig. 6) across different “target coverage rates”. The model achieved a reduction rate of 7.30% of Hgb tests at a “target coverage rate” of 0.90 (S Table 3). However, the reduction rate did not increase monotonically as the “target coverage rate” increased from 0.90 to 0.95, indicating that the model’s confidence may not accurately represent a different cohort at a large coverage rate. When the target coverage rate was over 0.9, the model achieved higher accuracy on MIMIC III data, implying that the overall MIMIC III data may have higher predictability in estimating Hgb values. Conversely, for a “target coverage rate” <  = 0.9, the model obtained lower accuracy on MIMIC III data because it selected more samples (e.g., 89.78% selected samples at “target coverage rate” of 0.75, S Table 3) with lower prediction confidences. While some normality AUCs and AUPRCs did not drop steadily, stability AUCs and AUPRCs decreased as the “target coverage rate” increased, suggesting that the model was particularly robust at detecting stable Hgbs. The trends in value performance were similar to those observed with local hospital data (S Fig. 6).

## Discussion

We introduced a deep learning model with a selective framework to address the laboratory reduction problem. We conducted a case study on Hgb reduction. Based on the definition that unnecessary laboratory tests are the ones predicted to be normal and stable, we demonstrated that our selective model achieved good predictive performance. Our major contribution is to offer safe recommendations for omitting unnecessary Hgb samples by jointly considering the confidence and prediction accuracy during training and testing. The idea was to select a proportion of Hgb candidates with high prediction confidence in estimating normality and stability. Our model automatically identified an appropriate balance between stability, normality, and prediction confidences, which achieved a performance of 95.89% normality AUC and 95.94% stability AUC with the potential to eliminate 9.91% of Hgb tests. In addition, when future Hgb tests were predicted as normal, our model predicted > 90% of the corresponding predicted Hgb values within a tolerable error range of 3% at a 90% selection coverage, demonstrating its robustness.

We also made some technical contributions in order to handle irregular time sequences in laboratory reduction by introducing a feed-forward attention function to capture the importance of every timestep. The ablation study results confirmed that both selective mechanism and attention-based LSTM layers contributed to the improvement of model performances. The model achieved competitive accuracy, even with the lower prediction confidence, when externally validated with ICU patients in the MIMIC III database. The high accuracy in external evaluation suggests that our model generalized well to ICU patients at different health institutions.

Our model allows for the integration of a confidence-based selection process into the training and testing phase. While decision trees and random forests can incorporate measures of uncertainty in the form of node impurity or feature importance scores during the training phase, they lack measures of confidence in the form of prediction outcomes. In contrast, our proposed approach estimates prediction confidence directly from the model’s output probabilities and uses it to select samples with high reliability for recommendations. By doing so, the model can focus on the most informative samples while minimizing the risk of false positives or false negatives. This makes our approach appropriate to the specific problem of identifying unnecessary laboratory tests and making safe reduction recommendations.

Not all predictable laboratory tests are unnecessary in complicated clinical situations. To ensure usability, our model discovered unnecessary Hgb tests among predicted high-confidence candidates. The predicted low-confidence Hgb samples are not recommended for elimination, for which our model would acknowledge ‘I don’t know’ and let clinicians decide. Our model also offers a tunable parameter to control the confidence level for sample inclusion (i.e., expected selection coverage). Clinicians can choose a confidence level based on the context to receive recommendations for clinical decision making. For some severe patients, the desired confidence level might be lifted to a high level such that we would reduce fewer laboratory tests to more closely monitor the patient. Since our approach was designed to identify unnecessary laboratory tests in the routine laboratory monitoring, it may not identify certain situations such as surgical procedures or hemoptysis, in which a clinician orders a test based on observed clinical events. In situations where there are clinical observations or other factors that might indicate the need for a laboratory test, the clinician should use their judgment to decide whether the test is necessary, regardless of the predictions made by our approach.

## Conclusion

Although our model has shown promising results in identifying unnecessary Hgb tests among predicted high-confidence candidates, it has some limitations. First, the optimal expected coverage rate that controls the model’s confidence might vary by different situations, and it is not directly interpretable as confidence intervals that are familiar to clinicians. Second, when applied to external patients admitted to another health institution (MIMIC III), the model would reduce fewer Hgb samples to ensure the confidence guarantee. Third, our model recommends a reduction of an individual laboratory test. In clinical practice, Hgb tests are commonly ordered as a part of a complete blood count panel, which includes platelet count and white blood count. Our model currently does not account for bundled test reduction strategies because the laboratory panel information is missing in the dataset. Finally, the ground truth of unnecessary laboratory tests might not be perfect. Our model relies on the standard of laboratory normal ranges, whereas some abnormal results may be predictable and stable (e.g., a clinically stable patient with stable anemia) and thus could be omitted.

Our approach is applicable to any scenario where time series data is available and where the prediction of laboratory test results would be useful. While our study specifically focused on predicting Hgb tests, it could be generalized to platelet count (Plt) and white blood count (WBC). It is because most clinical cases focus on dropping values of these laboratory tests, making it appropriate to use a similar definition of normality and stability. Ideally, the general approach we developed can be adapted to other laboratory tests with different definitions of normality and stability (e.g., including the case with increasing values) by replacing the input features and output labels with those specific to the new test. For example, if we were interested in predicting glucose levels, we would use glucose-specific features and gold-standard labels.

Future research will address these limitations and further explore the applicability of our model to other laboratory tests, as well as its integration into clinical decision-making process.

## Supplementary Information

**Additional file 1.**

## Data Availability

These clinical data contain potentially identifying and sensitive patient information. Even though the study has IRB approval, we cannot made patient data available due to ethical concerns and the privacy protection policy of the hospital. Custom code used to analyze the model is available upon reasonable request to the corresponding author.
