# Supplementary material for: Confidence-based laboratory test reduction recommendation algorithm
Source: BMC Med Inform Decis Mak. 2023 May 10;23:93. doi: 10.1186/s12911-023-02187-3 (PMC10173656; doi:10.1186/s12911-023-02187-3)
Supplement: Supplementary file 1 — Additional file 1. [file 12911_2023_2187_MOESM1_ESM.docx]

# Supplementary Information

## Supplementary Notes

### Data analysis

Statistical analyses were conducted in Python (version 3.7). The network model is processed via PyTorch libraries using CUDA. The network was trained using the Adam optimizer, with a 64 minibatch size, an L2 regularization weight of 0.000001, and a learning rate of 0.0001 over 100 epochs. The network hyperparameters producing the lowest total loss in the test set were chosen as the final network architecture.

### Relative Positional Time Embedding

The laboratory test data are represented as irregular time series, which have wide and heterogeneous time gaps between consecutive observations. The number of hour differences from the last laboratory draw contains positional information that indicates the relative relationships between lab test values in consequent blood draws. The time differences were concatenated with other input features (i.e., laboratory values, vital signs, patient demographics, Hgb value changes, and observation indicators) in the timestamp. Previous work [1] handled irregular time-series predictions by assigning the absolute time difference to each timestep. We assigned a unique temporal encoding to cover all possible hour gaps (relative differences by hours), which can be generalized to both short and long time gaps. Following the transformer model[2], we used sinusoidal functions to incorporate relative positions.

| $S(\triangle t)^{2i}=sin(\triangle t/10000^{2i/d})$ | (1) |
| --- | --- |
| $S(\triangle t)^{2i+1}=cos(\triangle t/10000^{(2i+1)/d})$ | (2) |

where $\triangle t$ is the relative time differences of laboratory tests, $d$ represents the embedding dimension, and $S:N->R^{d}$ is the sinusoidal function that produces the output vector for each dimension $i$. This kind of embedding allows us to represent relative positions, in which $S(t+k)$ is a linear transformation of $S(t)$ for any fixed offset $k$.

**Supplementary Results**

### Performance on Clinical Heterogeneity

We conducted an analysis to illustrate the relationship between model accuracy and patient characteristics or reduction policies. For gender subgroups, the results (**S Fig 1.a**) indicated that females achieved marginally higher accuracy in Hgb normality and stability than males. This is because females constitute the majority population in our dataset, making them more likely to be selected as high-confidence candidates by the model. However, for race subgroups, the results (**S Fig 1.b**) revealed that black patients achieved higher accuracy in Hgb normality and stability, despite being one of the minority populations. The model performance for race subgroups might be impacted by patient-specific information, such as disease history or medication usage. Therefore, one approach to mitigate the gender and race disparity is to fine-tune the model on those underrepresented subsets to improve its performance.  Another approach is to incorporate additional patient-specific features that might help the model better capture the underlying patient characteristics.

Regarding reduction policies, we evaluated the proposed model's performance when laboratory reduction started at different timestamps. The starting time of laboratory reduction at timestamp $t$ means that the model learned past observations prior to timestamp $t$, then started to corrupt observations that were predicted to be reduced at and after timestamp $t$. A prior study [3] of unnecessary laboratory tests has indicated that machine learning models could achieve poor performance at the last observation timestamps. This is because few encounters have conducted laboratory tests for more than 20 times (**S Fig 3**), making these encounter subsets underrepresented in machine learning models. To address the issue, we evaluated the model under different reduction policies to recognize whether the proposed model could improve performance if it captured more historical information. Our results (**S Fig 2**) revealed that the accuracy of Hgb normality and stability improved as the starting time of laboratory reduction increased, indicating the importance of enriched historical data. Although the accuracy decreased after timestamp $t=20$ for Hgb normality and $t=15$ for Hgb stability, their accuracy was still higher than those at the initial timestamp $t=0$. Overall, the evaluation results suggested that starting to perform reduction at an intermediate timestamp could mitigate poor performance of encounters with long observations.

## Supplementary Figure

1. **Model Accuracy and Gender Subgroups**

| 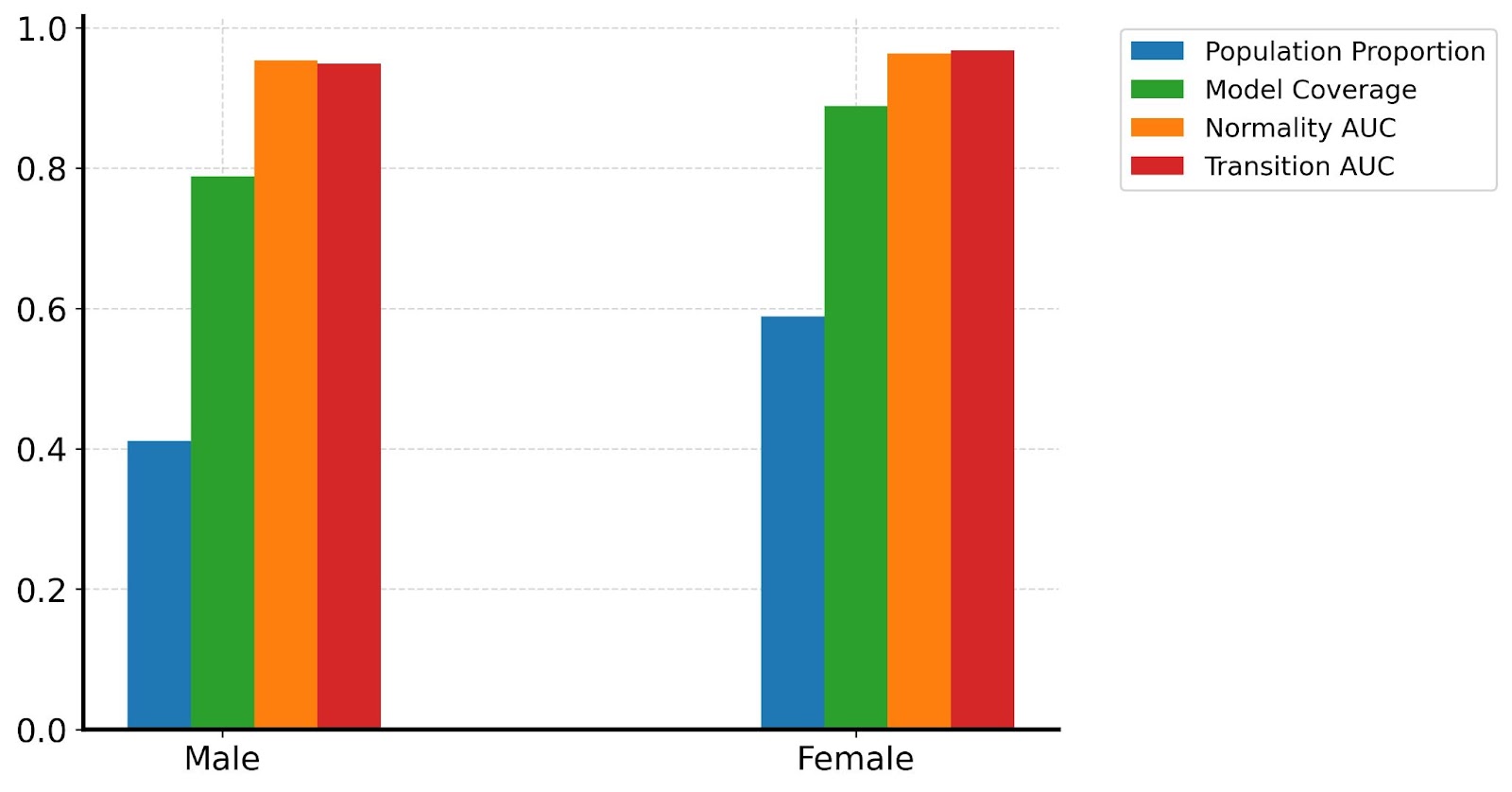 |
| --- |
| 1. **Model Accuracy and Race Subgroups**   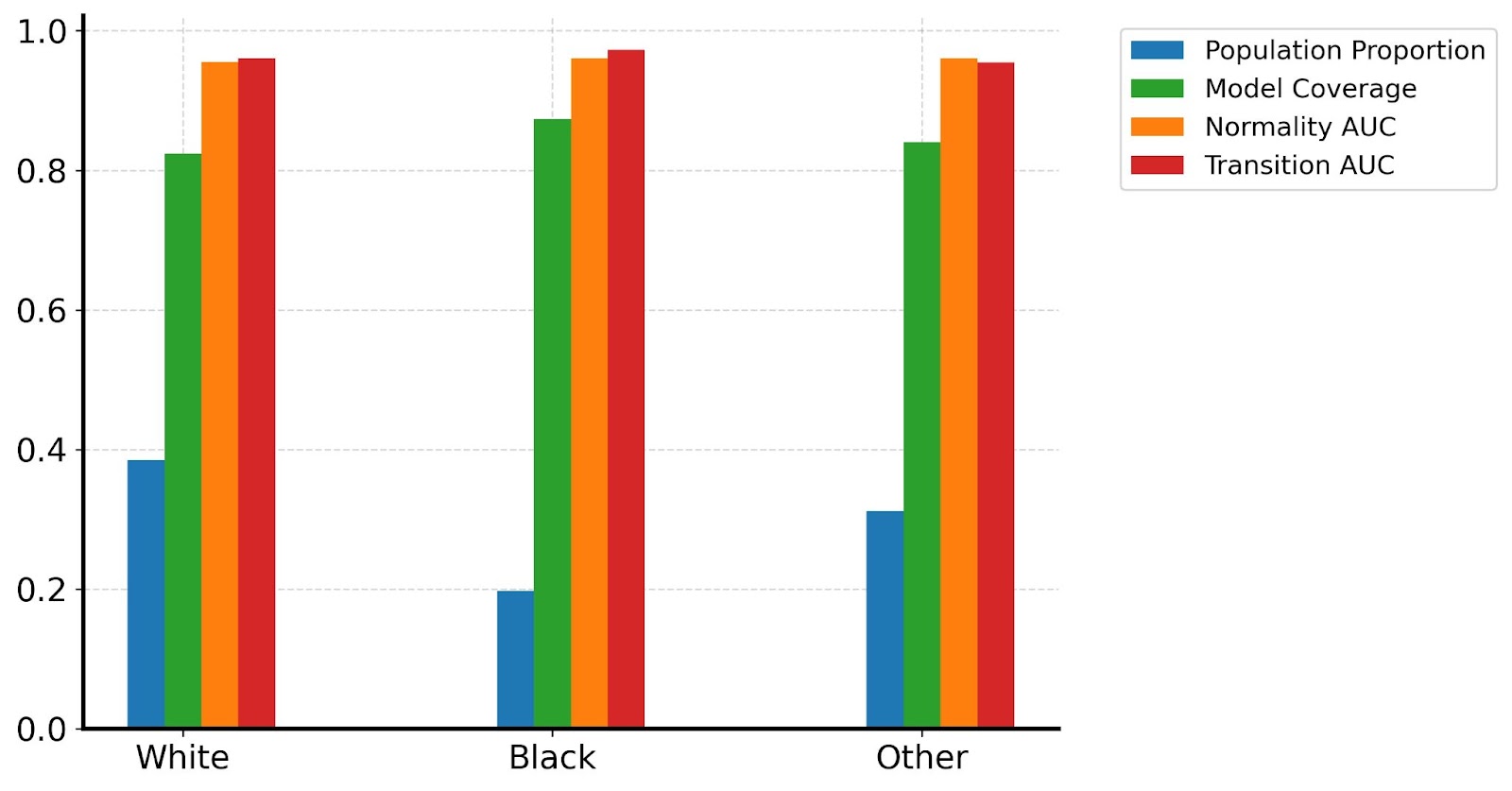 |

**S Fig 1. The relation between model accuracy and patient characteristics.**

| 1. **Normality AUC and reduction policies**   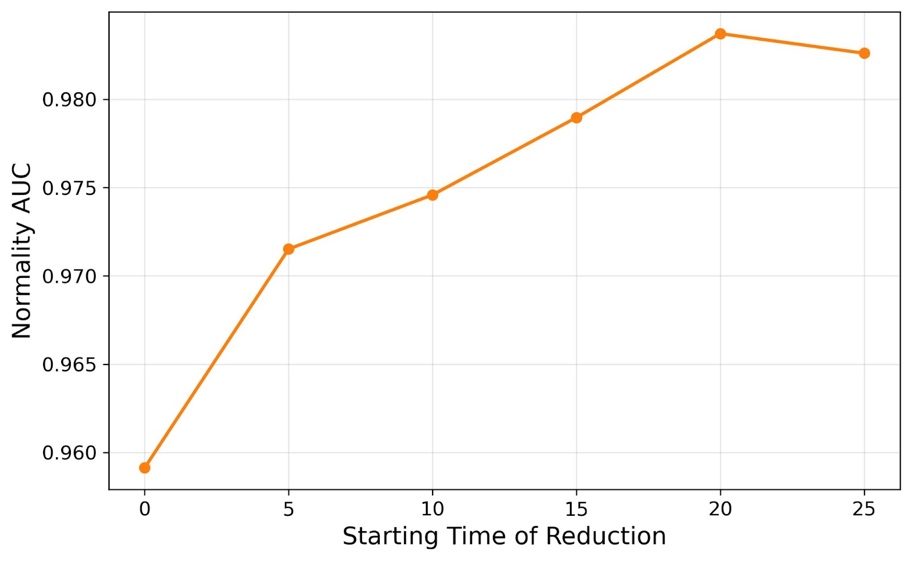 | 1. **Stability AUC and reduction policies**   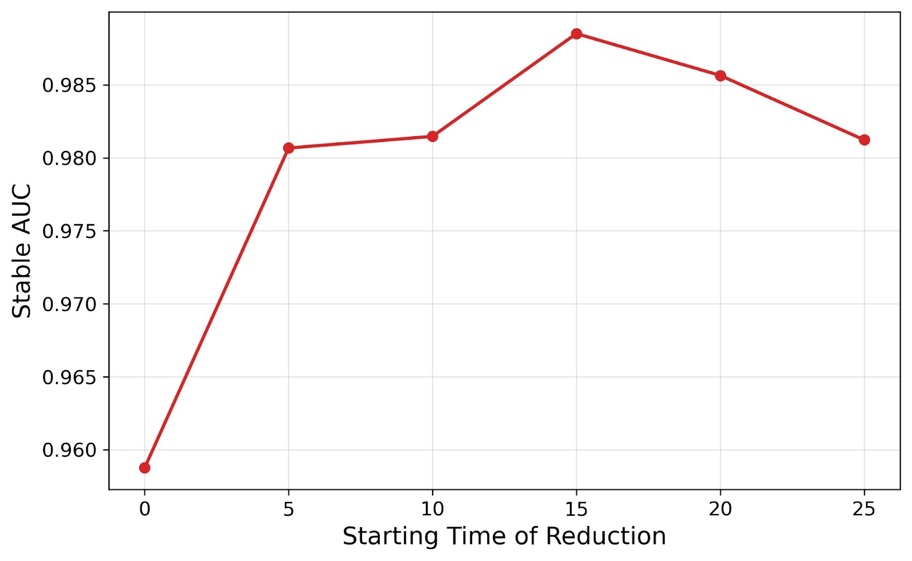 |
| --- | --- |

**S Fig 2. The relation between model accuracy and reduction policies.**


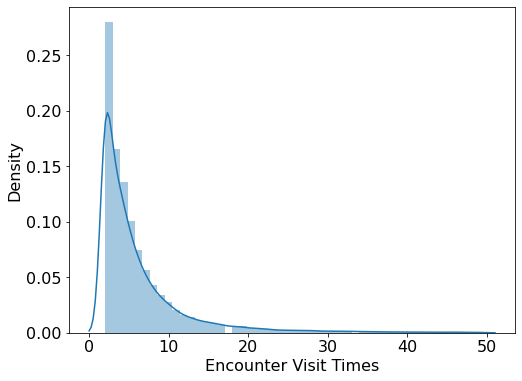


**S Fig 3. Histogram of encounter visit times.** Encounter visit times refer to the number of laboratory draws for each encounter. The histogram results show that it follows a long tail distribution. Most encounters have no more than 30 laboratory test records.


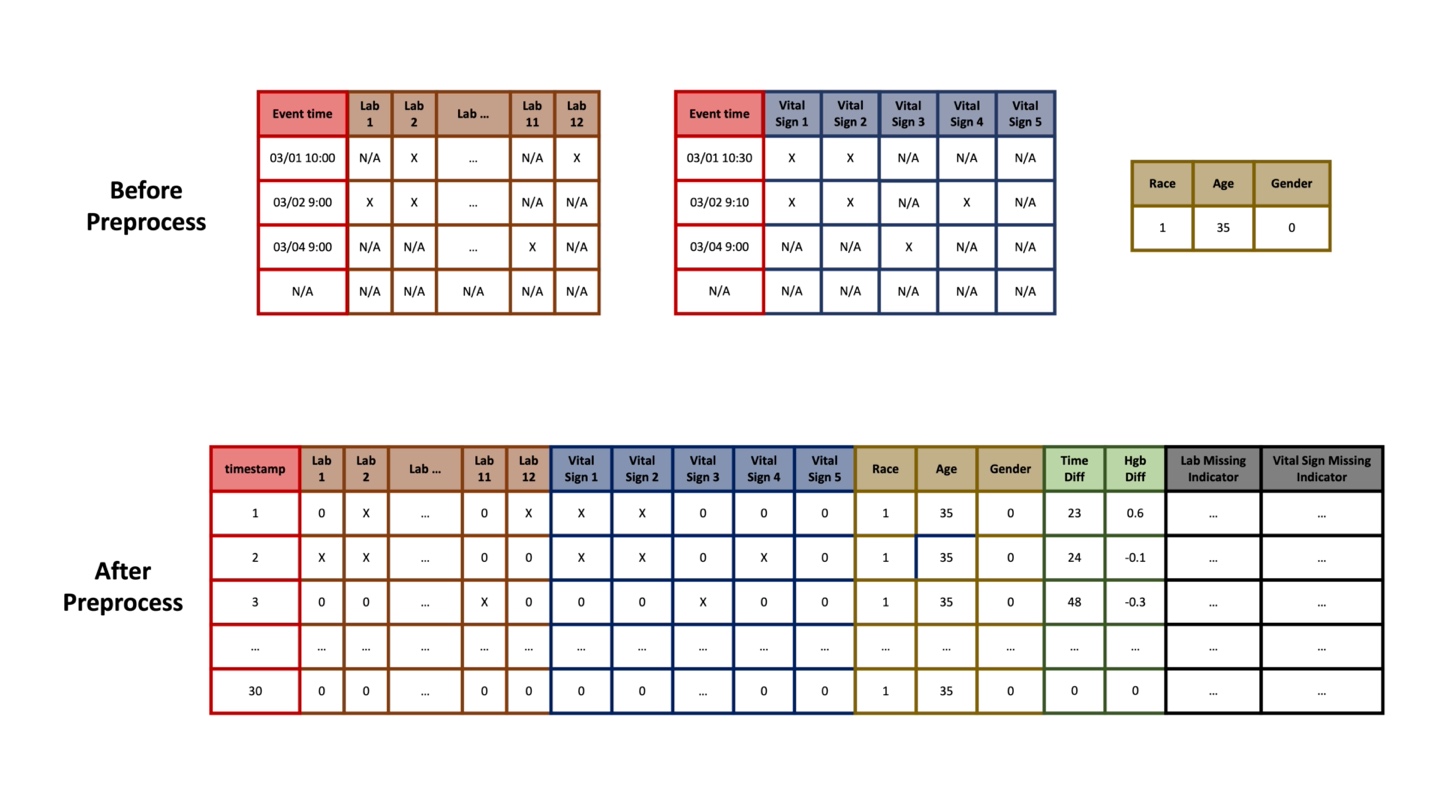


**S Fig 4. The structure of the dataset before and after processing.** The input features of an individual encounter consist of laboratory tests, vital signs, demographics, time differences (i.e., time from last observation), Hgb value changes, and missing value indicators. Before data preprocessing, laboratory tests and vital signs were recorded in two separated tables, with each event time linked to laboratory tests or vital signs that were performed concurrently. There are missing values (denoted as N/A) due to the fact that no patients had all laboratory tests or vital signs performed at the same time. The encounter has three demographic information: race, age, and gender, with race and gender represented as categorial numbers. After data preprocessing, laboratory tests and vital signs that were performed within the same hour were combined into the same timestamp. The length of timestamps was capped to 30. Demographic information were duplicated 30 times in order to align with each timestamp. Time differences and Hgb value changes were calculated based on the sequential order of preprocessed observations.

| **a. Normality prevalence**  **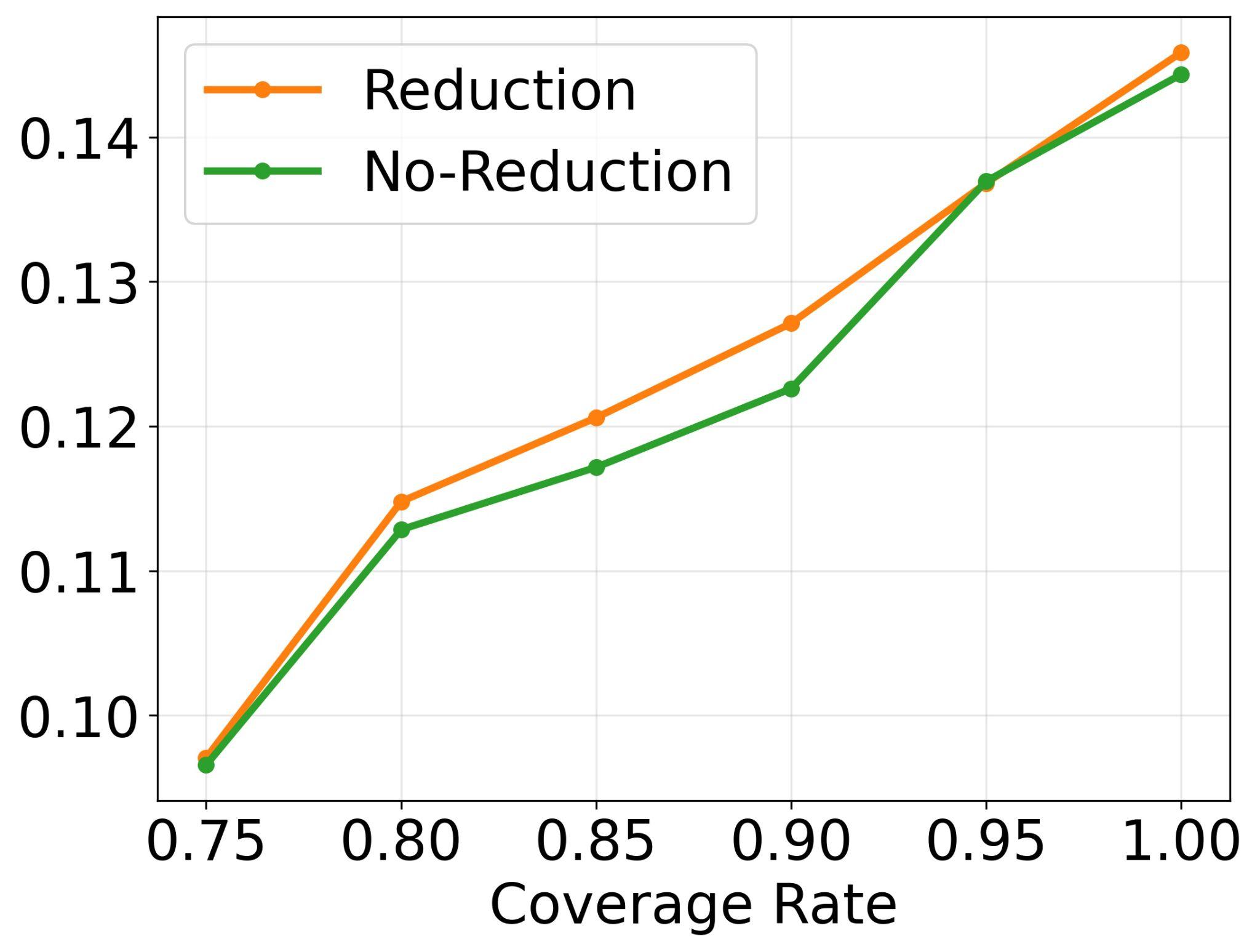** | **b. Normality AUC**  **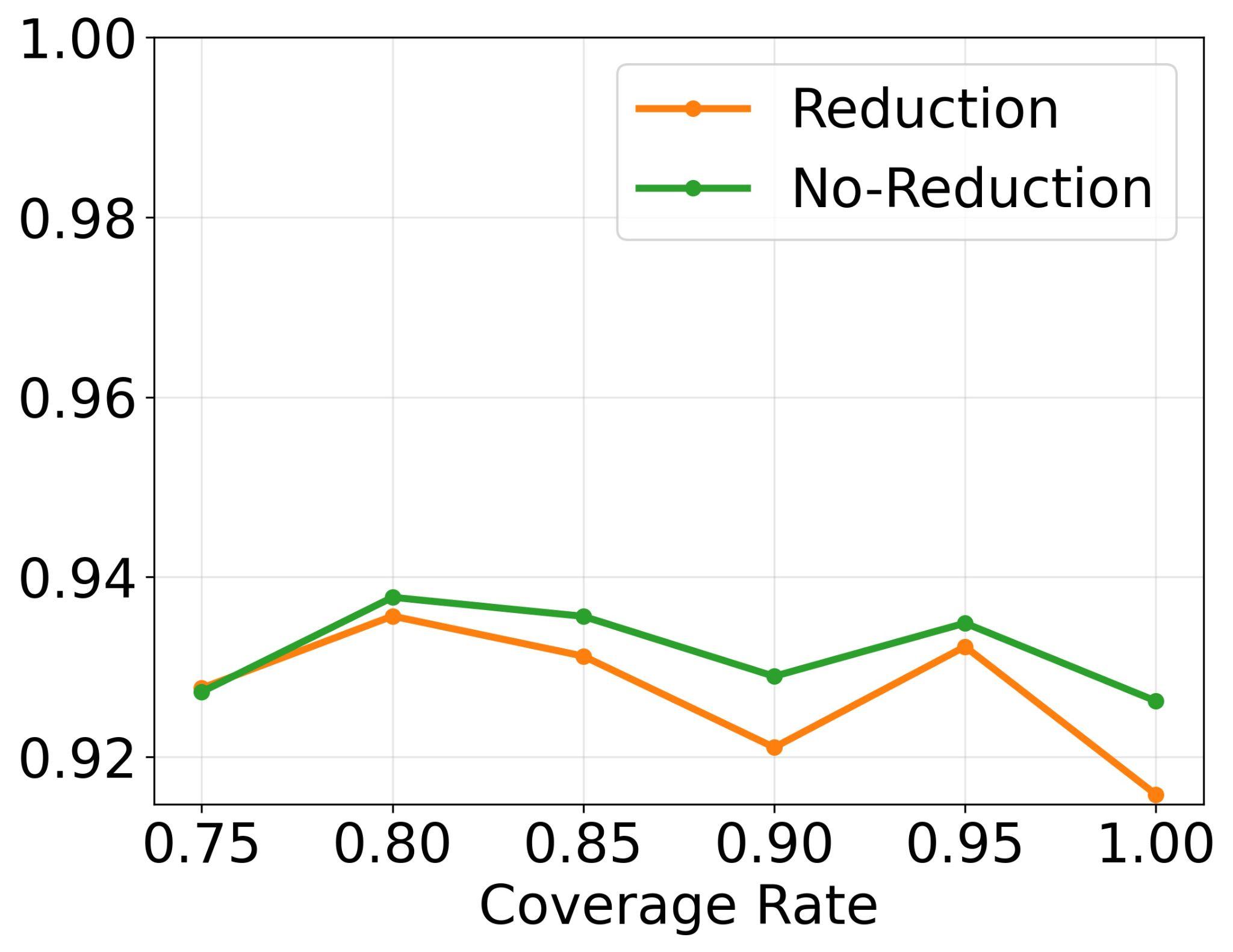** | **c. Normality AUPRC**  **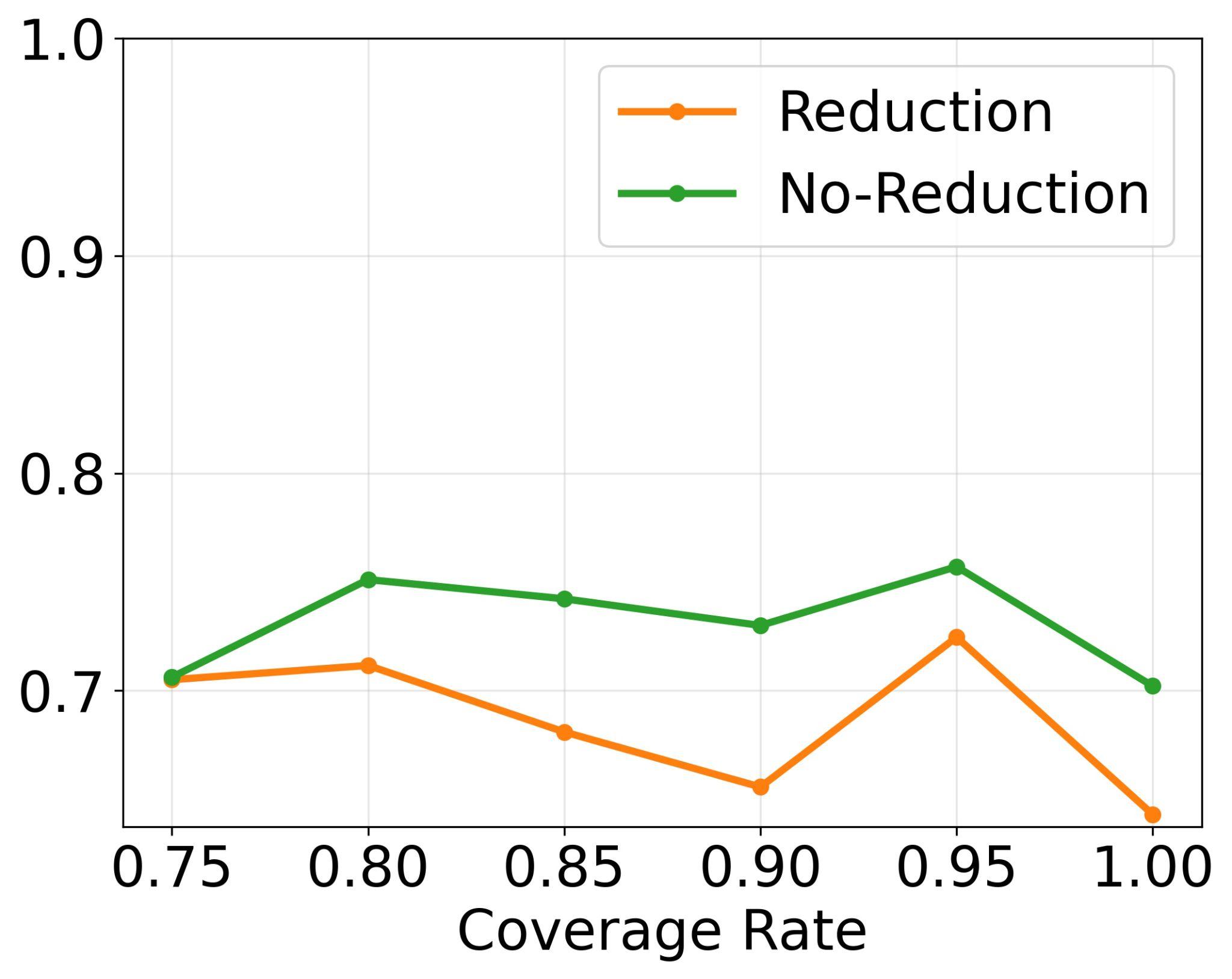** |
| --- | --- | --- |
| **d. Stability prevalence**  **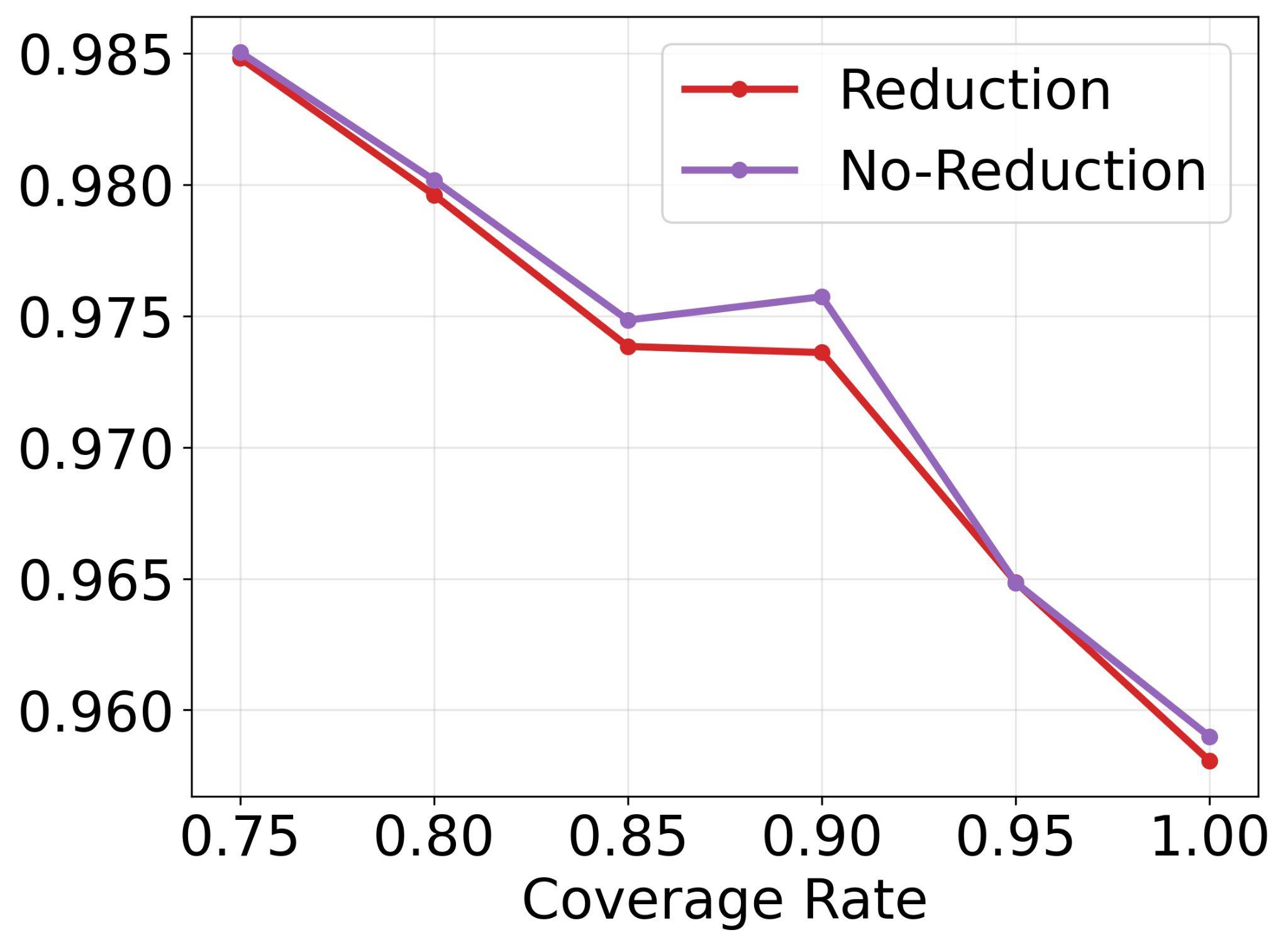** | **e. Stability AUC**  **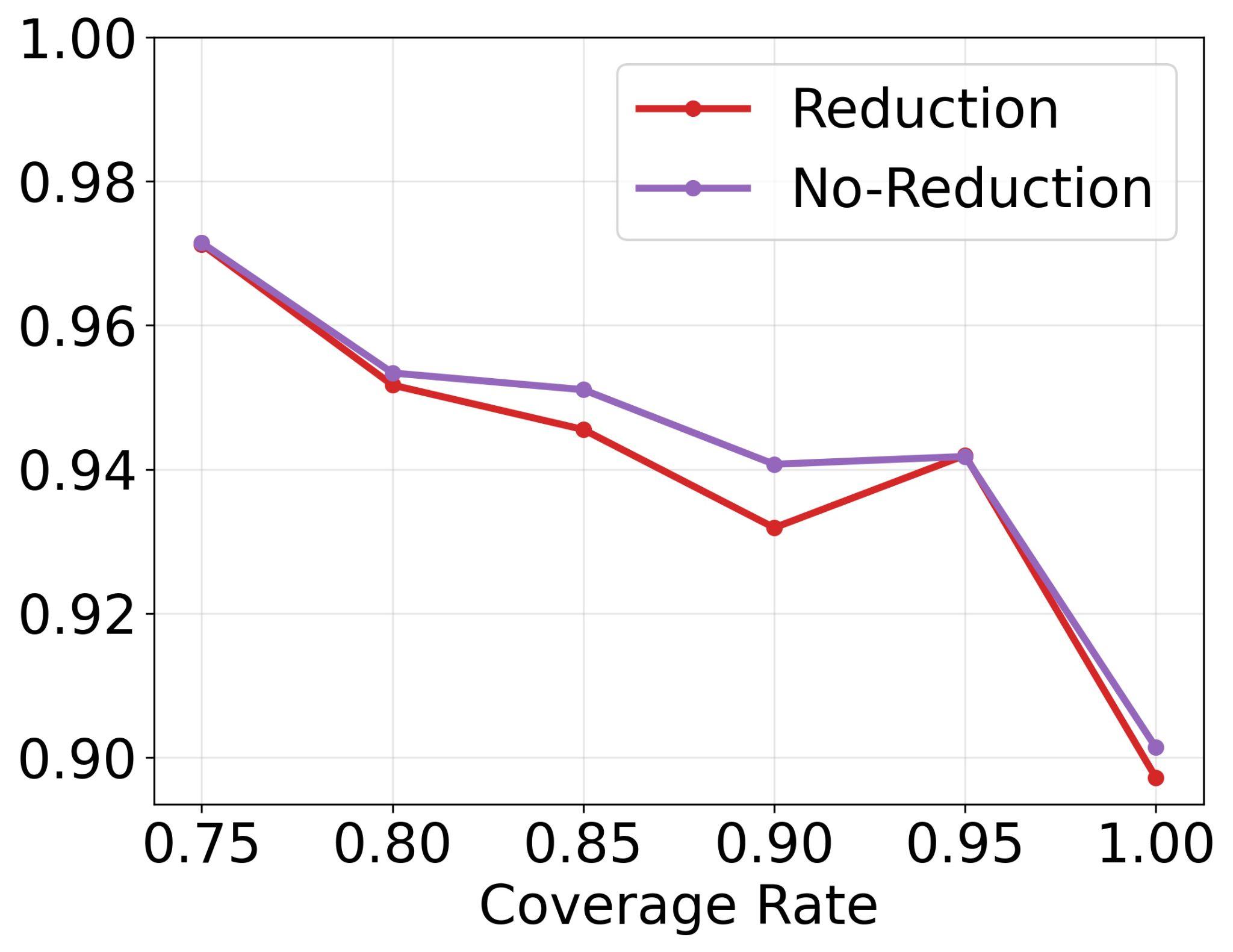** | **f. Stability AUPRC**  **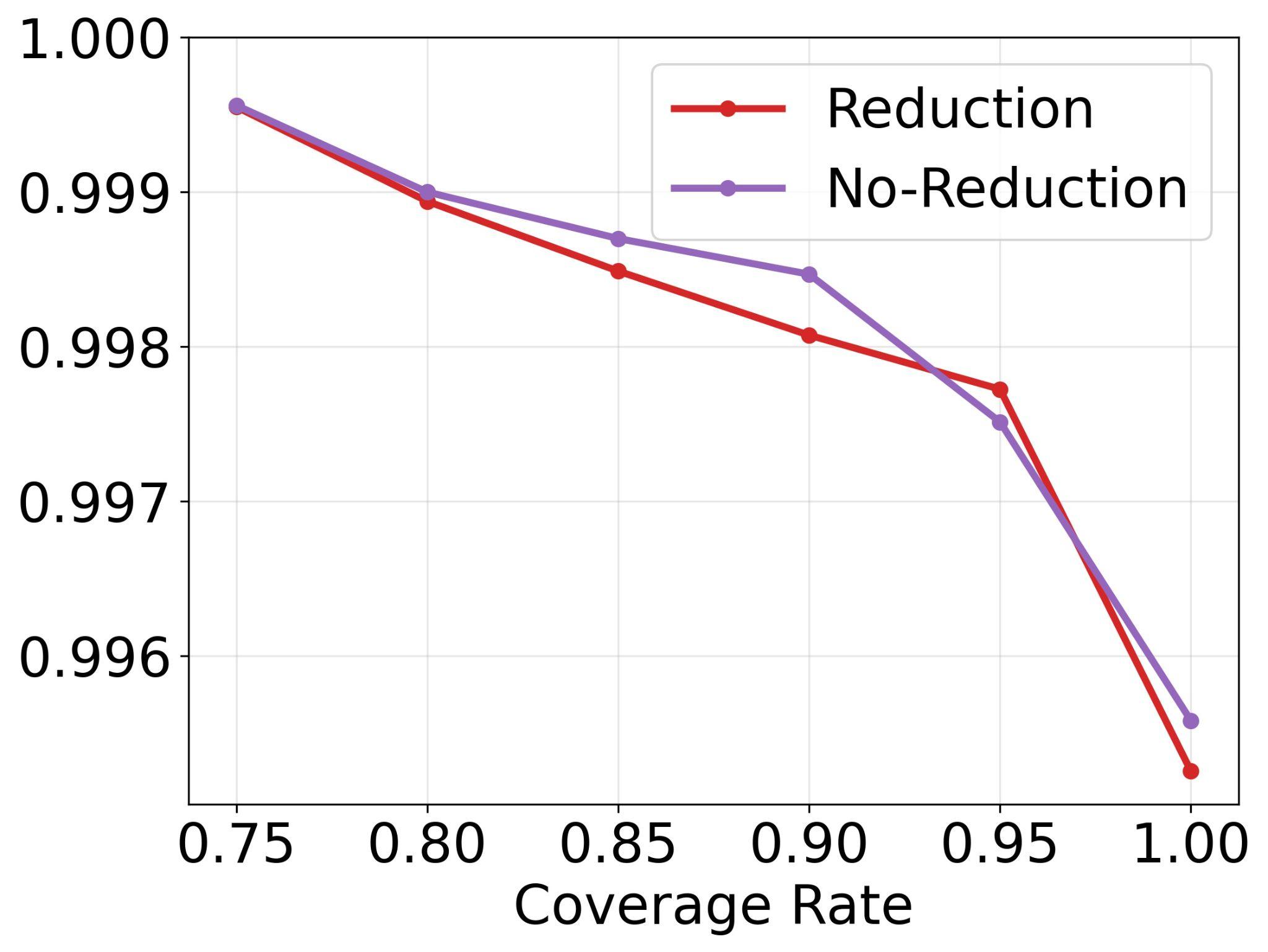** |

**S Fig 5. External validation results at multiple selection coverages under reduction and no-reduction evaluation.**


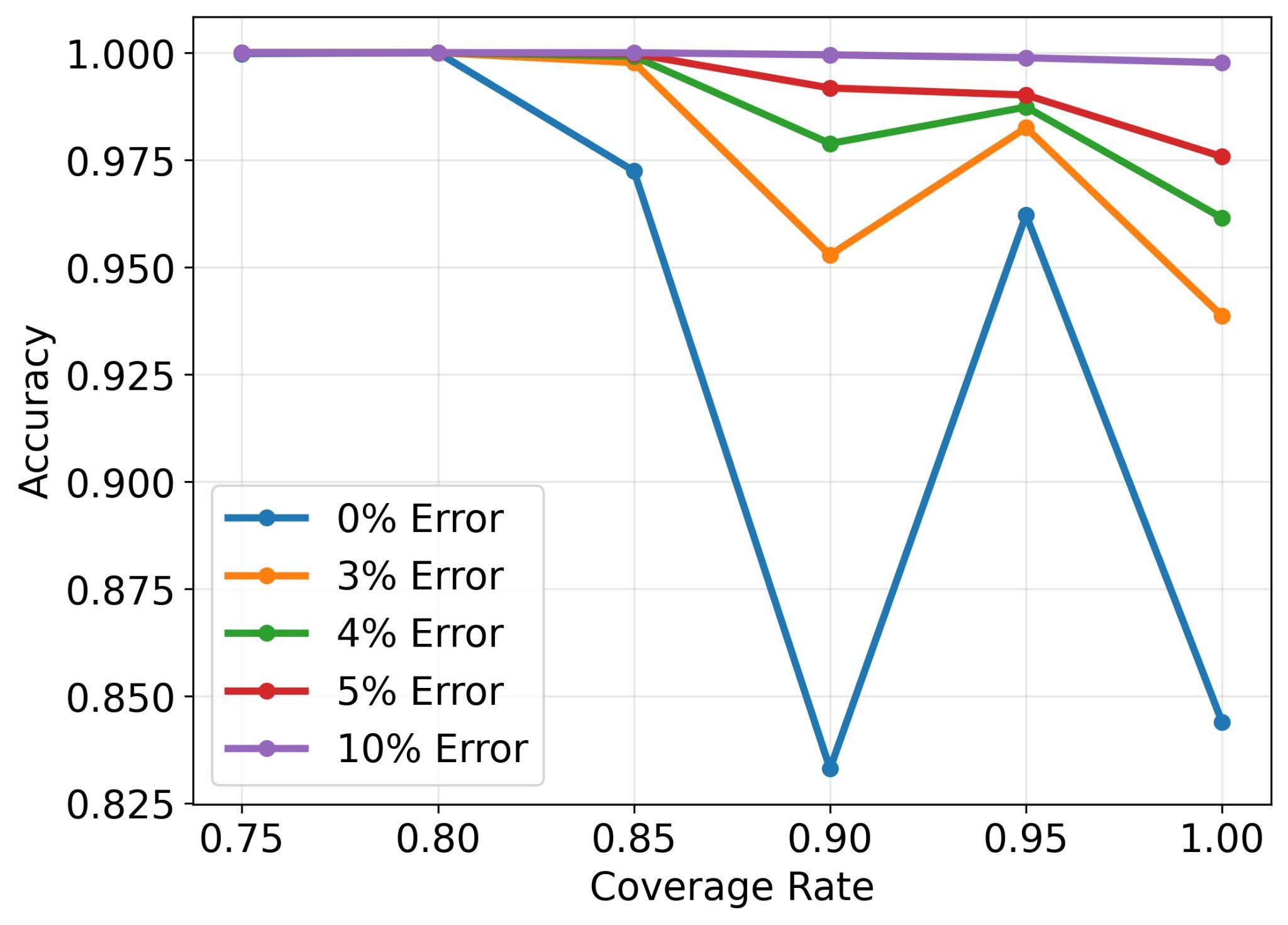


**S Fig 6. Consistency between predicted values and predicted normality, validated on external data.**

##

## Supplementary Tables

**S Table 1. Patient cohorts summarized.** This table includes input features of 3 patient demographic information (age, gender, and race), 5 vital signs, and 12 common laboratory tests.

|  | **MHHS Cohort**  **N = 62,479** | **MIMIC III Cohort**  **N = 46,847** |
| --- | --- | --- |
| **Demographics** |  |  |
| Age, year, mean | 54.02 (0-119) | 62.11 (0-90) |
| Male, n (%) | 25,707 (41.15%) | 26,409 (56.37%) |
| White | 24,032 (51.30%) | 33,533 (71.58%) |
| Black or African American | 12,336 (26.33%) | 4,532 (9.67%) |
| Asian | 2,045 (4.37%) | 1,144 (2.44%) |
| Other | 24,066 (38.52%) | 7,598 (16.23%) |
| **Vital Signs** |  |  |
| Peripheral pulse rate, mean (sd) | 84.06 (18.57) | 93.29 (26.78) |
| Respiratory rate, mean (sd) | 20.01 (5.84) | 19.32 (5.99) |
| SpO2 percent, mean (sd) | 96.77 (3.16) | 97.27 (3.32) |
| Diastolic blood pressure, mean (sd) | 73.47 (13.89) | 62.97 (308.84) |
| Systolic blood pressure, mean (sd) | 130.89 (22.61) | 124.94 (21.98) |
| **Laboratory Values** |  |  |
| Normal Hgb value (%) | 24.74% | 17.34% |
| Stable Hgb* (%) | 90.80% | 95.29% |
| BUN, mean (sd) | 24.42 (20.19) | 28.02 (22.19) |
| Calcium, mean (sd) | 8.60 (0.72) | 8.39 (0.78) |
| Chloride, mean (sd) | 105.22 (6.23) | 103.98 (6.14) |
| Creatinine, mean (sd) | 1.56 (1.91) | 1.50 (1.59) |
| HCO₃, mean (sd) | 26.56 (7.01) | 25.41 (4.91) |
| Hgb, mean (sd) | 11.26 (2.20) | 10.37 (1.77) |
| Magnesium, mean (sd) | 2.15 (0.46) | 2.04 (0.42) |
| Phosphorus, mean (sd) | 3.39 (1.25) | 3.53 (1.31) |
| Platelet, mean (sd) | 246.71 (118.36) | 236.47 (106.09) |
| Potassium, mean (sd) | 3.98 (0.60) | 4.09 (0.61) |
| Sodium, mean (sd) | 138.46 (4.94) | 138.69 (4.95) |
| WBC, mean (sd) | 9.88 (5.68) | 11.13 (8.80) |
| Sd – standard deviation. BUN – blood urea nitrogen. HCO₃ – sodium bicarbonate. Hgb – hemoglobin. SpO₂ – percent saturation of oxygenation. WBC – white blood cell count.  * Labs that did not change from normal to abnormal. | |  |

**S Table 2. The number of Hgb coverages and reduction rates under reduction evaluation (local hospital data).** The total number of Hgb samples in the test dataset is 57,039 starting from $t=0$, and 44,586 starting from $t=1$. The dominator of the coverage rate and the reduction rate is the total number of Hgbs starting from $t=1$, which ignores initial tests at $t=0$.

| **Target Coverage** | **Model Coverage** | **Covered Hgb Counts** | **Reduction Rate** | **Reduced Hgb Counts** |
| --- | --- | --- | --- | --- |
| **0.75** | 77.92% | 34,741 | 3.08% | 1,373 |
| **0.80** | 81.64% | 36,400 | 4.94% | 2,201 |
| **0.85** | 83.80% | 37,363 | 9.91% | 4,420 |
| **0.90** | 89.84% | 40,056 | 11.71% | 5,221 |
| **0.95** | 96.91% | 43,210 | 16.89% | 7,530 |
| **1.0** | 99.99% | 44,583 | 17.48% | 7,793 |

**S Table 3. The number of Hgb coverages and reduction rates under reduction evaluation (MIMIC III data).** The total number of Hgb samples in the test dataset is 429,614 starting from $t=0$, and 382,767 starting from $t=1$. The dominator of the coverage rate and the reduction rate is the total number of Hgbs starting from $t=1$, which ignores initial tests at $t=0$.

| **Target Coverage** | **Model Coverage** | **Covered Hgb Counts** | **Reduction Rate** | **Reduced Hgb Counts** |
| --- | --- | --- | --- | --- |
| **0.75** | 89.78% | 343,654 | 0.87% | 3,331 |
| **0.80** | 91.60% | 350,615 | 2.55% | 9,767 |
| **0.85** | 93.69% | 358,609 | 4.13% | 15,798 |
| **0.90** | 93.93% | 359,551 | 7.39% | 28,275 |
| **0.95** | 96.46% | 369,221 | 4.00% | 15,294 |
| **1.0** | 97.89% | 374,672 | 9.02% | 34,539 |

**S Table 4. Model performance under selection coverages under no-reduction evaluation (local hospital data).**

| **Target Coverage** | **Model Coverage** |  | **Normality** | | | | |  | **Stability** | | | | |
| --- | --- | --- | --- | --- | --- | --- | --- | --- | --- | --- | --- | --- | --- |
|  |  |  | **Prev** | **AUC** | **Acc** | **Prec** | **AUPRC** |  | **Prev** | **AUC** | **Acc** | **Prec** | **AUPRC** |
| **0.75** | 79.75% |  | 11.59% | 96.46% | 95.48% | 85.19% | 85.86% |  | 99.68% | 95.88% | 99.68% | 99.68% | 99.99% |
| **0.80** | 83.05% |  | 13.33% | 95.85% | 94.96% | 87.38% | 84.72% |  | 99.06% | 95.69% | 99.08% | 99.09% | 99.96% |
| **0.85** | 86.99% |  | 17.18% | 96.39% | 93.85% | 80.36% | 84.80% |  | 97.29% | 96.37% | 97.31% | 97.35% | 99.90% |
| **0.90** | 90.96% |  | 18.21% | 95.14% | 91.65% | 72.81% | 78.10% |  | 94.20% | 96.87% | 95.09% | 97.55% | 99.80% |
| **0.95** | 96.58% |  | 19.00% | 94.39% | 89.88% | 69.31% | 77.37% |  | 92.52% | 96.70% | 94.54% | 96.23% | 99.73% |
| **1.0** | 99.99% |  | 19.51% | 93.44% | 88.53% | 66.23% | 74.14% |  | 90.82% | 96.30% | 93.46% | 95.64% | 99.62% |

#

**S Table 5.** **Model performance by selection coverages and reduction rate (MIMIC III data).**

| **Target Coverage** | **Model Coverage** | **Reduction Rate** |  | **Normality** | | | | |  | **Stability** | | | | |
| --- | --- | --- | --- | --- | --- | --- | --- | --- | --- | --- | --- | --- | --- | --- |
|  |  |  |  | **Prev** | **AUC** | **Acc** | **Prec** | **AUPRC** |  | **Prev** | **AUC** | **Acc** | **Prec** | **AUPRC** |
| **0.75** | 77.92% | 3.08% |  | 9.74% | 92.79% | 91.48% | 93.74% | 70.55% |  | 98.47% | 97.12% | 99.48% | 98.51% | 99.95% |
| **0.80** | 81.64% | 4.94% |  | 11.48% | 93.55% | 90.84% | 79.80% | 71.14% |  | 97.95% | 95.19% | 97.96% | 97.96% | 99.89% |
| **0.85** | 83.80% | 9.91% |  | 12.07% | 93.12% | 90.93% | 73.75% | 68.13% |  | 97.39% | 94.53% | 97.39% | 97.39% | 99.85% |
| **0.90** | 89.84% | 11.71% |  | 12.71% | 92.11% | 90.48% | 65.86% | 65.64% |  | 97.36% | 93.17% | 97.22% | 97.43% | 99.80% |
| **0.95** | 96.91% | 16.89% |  | 13.74% | 93.40% | 88.97% | 81.53% | 71.45% |  | 96.52% | 94.39% | 96.53% | 96.65% | 99.79% |
| **1.0** | 99.99% | 17.48% |  | 14.59% | 91.58% | 88.05% | 60.94% | 64.32% |  | 95.81% | 89.71% | 95.30% | 96.02% | 99.53% |

**S Table 6. Model performance under selection coverages under no-reduction evaluation (MIMIC III data).**

| **Target Coverage** | **Model Coverage** |  | **Normality** | | | | |  | **Stability** | | | | |
| --- | --- | --- | --- | --- | --- | --- | --- | --- | --- | --- | --- | --- | --- |
|  |  |  | **Prev** | **AUC** | **Acc** | **Prec** | **AUPRC** |  | **Prev** | **AUC** | **Acc** | **Prec** | **AUPRC** |
| **0.75** | 89.72% |  | 9.66% | 92.72% | 91.58% | 95.69% | 70.58% |  | 98.50% | 97.14% | 98.51% | 98.54% | 99.96% |
| **0.80** | 91.43% |  | 11.29% | 93.78% | 90.73% | 93.15% | 75.10% |  | 98.01% | 95.33% | 98.01% | 98.02% | 99.90% |
| **0.85** | 93.29% |  | 11.71% | 93.56% | 91.10% | 89.08% | 74.18% |  | 97.45% | 95.11% | 97.49% | 97.49% | 99.87% |
| **0.90** | 93.22% |  | 12.26% | 92.90% | 91.87% | 78.42% | 73.03% |  | 97.57% | 94.06% | 97.44% | 97.65% | 99.85% |
| **0.95** | 96.57% |  | 13.62% | 93.67% | 89.02% | 90.80% | 74.16% |  | 96.55% | 94.71% | 96.56% | 96.68% | 99.80% |
| **1.0** | 97.70% |  | 14.44% | 92.62% | 89.09% | 72.04% | 70.22% |  | 95.90% | 90.12% | 95.38% | 96.13% | 99.56% |

#

**S Table 7. Value prediction consistency with normality under reduction evaluations.** This table provides numerical details on value prediction to explain **Fig 4**.

| **Target Coverage** | **Accuracy** | **Accuracy within 3% Error** | **Accuracy within 4% Error** | **Accuracy within 5% Error** | **Accuracy within 10% Error** |
| --- | --- | --- | --- | --- | --- |
| **0.75** | 88.32% | 95.57% | 96.78% | 97.52% | 99.63% |
| **0.80** | 89.86% | 97.97% | 99.05% | 99.47% | 99.89% |
| **0.85** | 76.15% | 93.52% | 95.91% | 97.50% | 99.86% |
| **0.90** | 65.74% | 83.24% | 87.62% | 90.94% | 99.03% |
| **0.95** | 58.49% | 79.90% | 85.30% | 89.28% | 98.40% |
| **1.0** | 50.88% | 81.09% | 87.16% | 91.22% | 99.07% |

**S Table 8. Model performances using different selection thresholds under reduction evaluation.** This evaluation table provides numerical details of selection prediction to explain **Fig 5**.

| **Selection Threshold** | **Model Coverage Rate** | **Normality** | | | **Stability** | | |
| --- | --- | --- | --- | --- | --- | --- | --- |
|  |  | **Prevalence** | **AUC** | **AUPRC** | **Prevalence** | **AUC** | **AUPRC** |
| 0.05 | 85.41% | 16.64% | 95.56% | 78.36% | 96.67% | 95.31% | 99.83% |
| 0.1 | 85.04% | 16.48% | 95.67% | 78.86% | 96.84% | 95.31% | 99.84% |
| 0.15 | 84.77% | 16.38% | 95.68% | 78.85% | 96.91% | 95.44% | 99.85% |
| 0.2 | 84.55% | 16.33% | 95.80% | 79.33% | 97.05% | 95.45% | 99.86% |
| 0.25 | 84.42% | 16.23% | 95.81% | 79.32% | 97.05% | 95.51% | 99.86% |
| 0.3 | 84.26% | 16.13% | 95.82% | 79.30% | 97.07% | 95.45% | 99.86% |
| 0.35 | 84.11% | 16.09% | 95.86% | 79.49% | 97.17% | 95.52% | 99.87% |
| 0.4 | 83.99% | 15.99% | 95.88% | 79.48% | 97.19% | 95.57% | 99.87% |
| 0.45 | 83.90% | 15.96% | 95.91% | 79.61% | 97.23% | 95.62% | 99.87% |
| 0.5 | 83.81% | 15.90% | 95.94% | 79.79% | 97.30% | 95.56% | 99.87% |
| 0.55 | 83.67% | 15.82% | 95.93% | 79.78% | 97.30% | 95.66% | 99.88% |
| 0.6 | 83.53% | 15.81% | 96.00% | 80.15% | 97.37% | 95.62% | 99.88% |
| 0.65 | 83.45% | 15.73% | 96.01% | 80.11% | 97.40% | 95.71% | 99.88% |
| 0.7 | 83.28% | 15.65% | 96.05% | 80.30% | 97.47% | 95.71% | 99.89% |
| 0.75 | 83.13% | 15.59% | 96.11% | 80.60% | 97.52% | 95.70% | 99.89% |
| 0.8 | 82.97% | 15.51% | 96.12% | 80.66% | 97.60% | 95.69% | 99.89% |
| 0.85 | 82.78% | 15.40% | 96.18% | 80.97% | 97.71% | 95.75% | 99.90% |
| 0.9 | 82.52% | 15.30% | 96.24% | 81.33% | 97.82% | 95.70% | 99.90% |
| 0.95 | 82.08% | 15.06% | 96.33% | 81.75% | 97.97% | 95.87% | 99.91% |

**References**

1 A deep learning solution to recommend laboratory reduction strategies in ICU. *Int J Med Inform* 2020;**144**:104282.

2 Vaswani A, Shazeer N, Parmar N, *et al.* Attention Is All You Need. 2017.http://arxiv.org/abs/1706.03762 (accessed 13 Oct 2021).

3 Yu L, Zhang Q, Bernstam EV, *et al.* Predict or draw blood: An integrated method to reduce lab tests. *J Biomed Inform* 2020;**104**:103394.
